# PIM‐1L Kinase Binds to and Inactivates SRPK1: A Biochemical and Molecular Dynamics Study

**DOI:** 10.1002/prot.26757

**Published:** 2024-10-27

**Authors:** Nastazia Lesgidou, Anastasia Koukiali, Eleni Nikolakaki, Thomas Giannakouros, Metaxia Vlassi

**Affiliations:** ^1^ Institute of Biosciences and Applications National Center for Scientific Research “Demokritos” Athens Greece; ^2^ Laboratory of Biochemistry, Department of Chemistry Aristotle University Thessaloniki Greece

**Keywords:** allosteric communications, cancer mutations, community map analysis, molecular dynamics simulations, PIM‐1 kinase, protein kinases, SR protein kinase 1

## Abstract

SR/RS dipeptide repeats vary in both length and position, and are phosphorylated by SR protein kinases (SRPKs). PIM‐1L, the long isoform of PIM‐1 kinase, the splicing of which has been implicated in acute myeloid leukemia, contains a domain that consists largely of repeating SR/RS and SH/HS dipeptides (SR/SH‐rich). In order to extend our knowledge on the specificity and cellular functions of SRPK1, here we investigate whether PIM‐1L could act as substrate of SRPK1 by a combination of biochemical and computational approaches. Our biochemical data showed that the SR/SH‐rich domain of PIM‐1L was able to associate with SRPK1, yet it could not act as a substrate but, instead, inactivated the kinase. In line with our biochemical data, molecular modeling followed by a microsecond‐scale all‐atom molecular dynamics (MD) simulation suggests that the SR/SH‐rich domain acts as a pseudo‐docking peptide that binds to the same acidic docking‐groove used in other SRPK1 interactions and induces inactive SRPK1 conformations. Comparative community network analysis of the MD trajectories, unraveled the dynamic architecture of apo SRPK1 and notable alterations of allosteric communications upon PIM‐1L peptide binding. This analysis also allowed us to identify key SRPK1 residues, including unique ones, with a pivotal role in mediating allosteric signal propagation within the kinase core. Interestingly, most of the identified amino acids correspond to cancer‐associated amino acid changes, validating our results. In total, this work provides insights not only on the details of SRPK1 inhibition by the PIM‐1L SR/SH‐domain, but also contributes to an in‐depth understanding of SRPK1 regulation.

## 1 Introduction

SRPKs constitute a subfamily of protein kinases highly conserved across eukaryotes that exert their functions by phosphorylating multiple serine residues residing in consecutive arginine‐serine dipeptide repeats, known as RS domains [1, 2]. SR proteins, as well as several other mammalian splicing factors, contain RS domains and for that reason, SRPKs are widely regarded as splice factor kinases involved in regulating various steps of mRNA splicing [3]. Yet, the mammalian genome contains more than a 100 RS domain‐containing proteins [4, 5, 6, 7], and SRPKs were progressively shown to exert pleiotropic functions via the phosphorylation of additional substrates and affect diverse cellular activities [1, 6, 7, 8].

Most of our knowledge regarding the phosphorylation mechanism used by SRPKs comes from studies on SRPK1. Phosphorylation by SRPK1 involves recognition of docking motifs in its substrates that are generally rich in basic residues, conforming to the consensus sequence R‐x‐R/K‐x(3)‐R. These docking motifs bind to an acidic groove of SRPK1, which is located far from the active site, and is known as docking groove [9, 10, 11, 12]. Although, initially, a common mechanism has been proposed to be used by the kinase to recognize its substrates, data from various reports indicate that the mechanism of phosphorylation is rather substrate‐dependent and may be influenced by the number of SR/RS repeats [10, 12, 13, 14].

There are few proteins in the literature that have been characterized as SRPK1 inhibitors and can be divided into two groups, depending on whether they interact with the kinase and block its activity or with the RS domains of the substrates, thus masking the phosphorylation sites. The first group contains proteins harboring RG/RGG motifs such as SAFB1/2, TAF15 and the viral protein ICP27. The ICP27 RGG box was shown to directly compete with RS domains for the docking groove of SRPK1, thus preventing phosphorylation of the substrates [15], while the C‐terminal domains of SAFB proteins and TAF15 that comprise the RGG/RG repeats were shown to bind to and inhibit SRPK1 [16, 17]. In addition, several peptides containing RG/RGG motifs were shown to function as potent SRPK1 inhibitors and most interestingly the observed inhibition was proportional to the number of RGG repeats [17]. The multifunctional cellular protein p32 is the most studied representative of the second group and has been reported to bind to the unmodified RS domains of ASF/SF2 and Lamin B receptor (LBR) and prevent their phosphorylation [18, 19].

Tzelepis et al. [20] have previously shown that SRPK1 is a key regulator in acute myeloid leukemia (AML). Genetic or pharmacological inhibition of SRPK1 led to leukemic cell differentiation and prolonged survival of mice transplanted with MLL‐rearranged AML. This was achieved via the alteration of isoform levels of many genes that were previously identified as cell‐essential for AML, including PIM‐1 (provirus integration site for Moloney murine leukemia virus 1) that encodes a Ser/Thr protein kinase. PIM‐1 is expressed primarily in B‐lymphoid and myeloid cell lines, and is overexpressed in hematopoietic malignancies and solid cancers [21]. Interestingly, it has been reported to encode two isoforms resulting from the use of alternative in‐frame translation initiation codons, an upstream non‐AUG (CUG) and a downstream AUG codon [22]. Thus, PIM‐1 is synthesized as long (PIM‐1L, 44 kDa) and short (PIM‐1S, 33 kDa) isoforms. However, they both start with a methionine, since early translation studies have shown that CUG codons can serve as initiators but they use the normal methionine initiator tRNA [22, 23]. Although very limited information is available about the substrates and the cellular function(s) of PIM‐1L, it is generally believed that the extra domain may allow PIM‐1L to interact with more proteins and crosstalk with more signaling networks as compared to PIM‐1S [22, 24]. Thus, even though both PIM‐1L and PIM‐1S exhibit comparable in vitro kinase activity, they may target distinct substrates.

**FIGURE 1:**
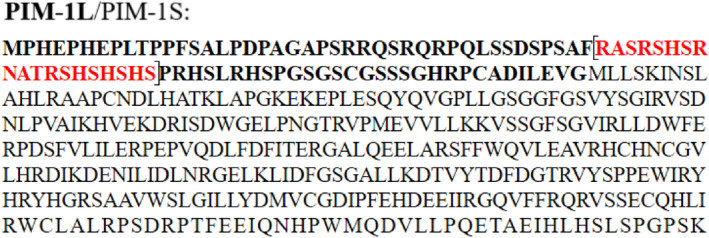
Amino acid sequence of PIM‐1L/PIM‐1S. The extra 91 amino acids at the N‐terminus of PIM‐1L, that are absent in PIM‐1S, are shown in bold. The SR/SH‐rich domain, which is removed in the PIM‐1LΔ construct studied in this work, is shown in red and in brackets.

Looking carefully at the extra N‐terminal sequence of PIM‐1L, we noticed the presence of an SR/SH‐rich domain which consists largely of repeating SR/RS and SH/HS dipeptides (Figure 1, in red). It is worth noting that the effect of substitution of histidine for arginine on the kinase activity has not been previously addressed. In this work, we thus sought to test whether PIM‐1L could act as a substrate of SRPK1. A combination of biochemical techniques and in silico approaches including microsecond‐scale (μs), all‐atom molecular dynamics (MD) simulations of SRPK1 in complex with a PIM‐1L peptide of the SR/SH domain as well as in its unbound form (termed APO SRPK1), followed by comparative community network analysis of the MD trajectories, were employed for this purpose.

Our biochemical data provided evidence that, although PIM‐1L was able to interact with SRPK1 and this interaction required its SR/SH‐rich domain, PIM‐1L is not an SRPK1 substrate but their interaction results, instead, in SRPK1 inhibition. On top of this observation, our MD data strongly suggested that the PIM‐1L SR/SH domain mimics the docking‐motif/docking‐groove interactions used by SRPK1 to recognize its substrates and/or inhibitors and that this interaction promotes inactive conformations of the kinase. We concluded that, PIM‐1L, through its SR/SH domain, may function as a pseudosubstrate and/or a pseudo‐docking peptide that binds to the same SRPK1 acidic docking‐groove used to recognize its substrates. Moreover, subsequent comparative community map analysis of the MD trajectories unraveled the details of the dynamic profile of the apo enzyme and shed light on alterations of allosteric signaling within SRPK1 in response to PIM‐1L peptide binding. Finally, and more importantly, this type of analysis pointed to key SRPK1 amino acids with a pivotal role in dynamics‐driven allosteric communications. Further validating our findings, the majority of these amino acids are related to cancer‐associated mutations of the *SRPK1* gene. In total, our results strongly suggest that the PIM‐1L SR/SH‐rich domain is not only involved in binding to the SRPK1 docking groove, but it severely affects allosteric signaling within the kinase domain (KD), leading to kinase inactivation.

## 2 Materials and Methods

### 2.1 Plasmids and Expression of Proteins

pGEX‐2T‐SRPK1 and pGEX‐2T‐LBRNt(62‐92) (expressing a 31‐amino‐acid fragment of the N‐terminal domain of LBR that contains the RS dipeptides) have been previously described [25].

Total RNA (1 μg), that was isolated from K562 cells using the TRIzol reagent, was converted to cDNA using M‐MLV Reverse Transcriptase (Invitrogen) and random hexamer primers, according to the manufacturer's recommendations. PIM‐1L cDNA was amplified with PCR from 10% of the first strand reaction (2 μL) with the upstream primer PIMsense 5′‐CCCGGGACTGCCGCACGAGCCC‐3′ (corresponding to the N‐terminal sequence of PIM‐1L and carrying an additional SmaI site at its 5′ end) and downstream primer PIMantisense 5′‐TGAATTCCTATTTGCTGGGCCCCGGCGACAG‐3′ (corresponding to the C‐terminal sequence of PIM‐1L and carrying an additional EcoRI site at its 3′ end) and ligated into the pGEM‐T Easy vector (Promega). The insert was then removed from pGEM‐T Easy, following digestion with SmaI/EcoRI and ligated into the SmaI/EcoRI site of pGEX‐2T.

To delete the SR/SH‐rich domain of PIM‐1L (43‐RASRSHSRNATRSHSHSHS‐61) and to obtain a PIM‐1L deletion construct (hereafter termed PIM‐1LΔ), 0.8 μg of PIM‐1L cDNA and two primers that were able to hybridize on either side of the deletion region (namely, delsense: 5′‐TCGCCCTCGGCCTTCCCCAGGCATAGCCTT‐3′; and delantisense: 5′‐AAGGCTATGCCTGGGGAAGGCCGAGGGCGA‐3′) were denaturated for 5 min at 95°C, left to hybridize for 1 h, then elongate for 30 min at 68°C, and finally amplified for 30 cycles using TaKaRa LA Taq polymerase. *E. coli* JM109 cells were transformed with the amplified DNA and 0.8 μg of the purified plasmid DNA obtained from all colonies was then subjected to two PCR reactions. In the first reaction, the primers used were PIMsense and delantisense; in the second, the primers were delsense and PIMantisense. PCR conditions were: denaturation at 94°C for 1 min, followed by 40 cycles: 30 s denaturation at 94°C, annealing and extension at 68°C for 35 s and 1 min, respectively, and a final extension at 72°C for 5 min. The obtained PCR fragments (141 bp from the first and 1047 bp from the second PCR reaction, respectively) were purified using the NucleoSpin Gel and PCR Clean‐up kit (Macherey‐Nagel). The two DNA segments (0.5 μg of each) were then denaturated for 5 min at 95°C and left to hybridize for 1 h. After hybridization, PIMsense and PIMantisense were added and PCR was carried out as follows: extension at 68°C for 30 min followed by 30 cycles: 30 s denaturation at 94°C, annealing and extension at 68°C for 35 s and 1 min, respectively, and a final extension at 72°C for 5 min. The amplified DNA was purified using the NucleoSpin Gel and PCR Clean‐up kit and ligated into the pGEM‐T Easy vector (Promega). Following transformation of JM109 cells, plasmid DNA was isolated from several colonies and the deletion was confirmed by sequencing at CEMIA SA (Larissa, Greece). The correct insert carrying the deletion was removed from pGEM‐T Easy, following digestion with SmaI/EcoRI and ligated into the SmaI/EcoRI site of pGEX‐2T.

The GST‐fusion proteins were produced in bacteria and purified using glutathione‐Sepharose (Amersham), according to the manufacturer's instruction.

### 2.2 Cell Culture

HeLa cells were cultured in DMEM medium supplemented with 10% (vol/vol) fetal bovine serum (FBS) and antibiotics (cell culture products were purchased from Gibco‐Invitrogen). Cells were incubated at 37°C with 5% CO_2_.

### 2.3 Pull‐Down Assays

Incubation of GST or GST‐PIM‐1L or GST‐PIM‐1LΔ (2–3 μg each) immobilized on glutathione‐Sepharose beads with HeLa cell extracts (∼200 μg of total protein) was performed in 50 mm Tris–HCl, pH 7.5, 150 mm NaCl, 1% Triton X‐100, and 0.5 mm PMSF (cell extraction buffer) in a total volume of 0.5 mL for 60 min at room temperature. Bound SRPK1 was analyzed on 10% SDS‐polyacrylamide gels and detected by Western blotting using the anti‐SRPK1 monoclonal antibody (611 072, BD Biosciences), an alkaline phosphatase‐coupled goat anti‐mouse secondary antibody, and 5‐bromo‐4‐chloro‐3‐indolyl phosphate/nitro blue tetrazolium substrate.

### 2.4 In Vitro Kinase Assays

Kinase assays were carried out at 30°C in a total volume of 25 μL containing 12 mM Hepes pH 7.5, 10 mM MgCl_2_, 25 μM ATP, 1–4 μg of the appropriate substrate (GST‐LBRNt(62‐92) or GST‐PIM‐1L) and 0.5 μg GST‐SRPK1. For the inhibition assays, GST‐SRPK1 was incubated with GST‐LBRNt(62‐92) under the assay conditions in the presence of increasing concentrations of GST‐ PIM‐1L or GST‐PIM‐1LΔ (5, 10, 15, and 20 μg). The samples were incubated for 30 min, and the reaction was stopped by adding 6 μL of 5 × SDS sample buffer and heating at 95°C for 3 min. Phosphoproteins were detected via autoradiography using Super RX (Fuji medical X‐ray film) and signals were quantified by excising the radioactive bands from the gel and Cherenkof counted. *p* Values were determined using a two‐tailed, unpaired student's *t*‐test.

### 2.5 Molecular Modeling/MD Simulations

#### 2.5.1 Construction of Initial 3D‐Model

A 3D‐model of the KD of SRPK1 in complex with a 9‐mer peptide of the SR/SH‐rich domain of PIM‐1L (aa: 42‐FRASRSHSR‐50) (Figure S1B), was constructed based on the known crystal structure of SRPK1‐KD (construct SRPK1ΔΝ1S1 in [11, 26], which also includes fragments of the spacer insert; namely, aa: 227–256 and 474–489) complexed with its substrate protein ASF/SF2 (PDB ID code: 3BEG [11]). Only the coordinates of SRPK1 and of the part of ASF/SF2 (aa: 202‐YGRSRSRSR‐210) that complies with the docking motif and is docked in the SRPK1 docking groove (Figure S1A) were kept as the modeling template and in silico mutagenesis through the graphics program PyMOL were used for this purpose. To minimize the possibility of salt‐bridge traps, ACE (acetyl) and NME (methylamine) blocking groups were used to cap the charged termini of the modeled PIM‐1L peptide. The produced 3D‐model was subsequently used as initial conformation for the MD simulation of the SRPK1/PIM‐1Lpept complex, whereas the coordinates of the SRPK1‐KD alone, were employed as starting conformation of the MD simulation of the SRPK1 APO form, for comparison.

#### 2.5.2 Molecular Dynamics Simulations

The all‐atom MD simulations were carried out in explicit water using GROMACS [27, 28]. For this purpose, the initial structures of both SRPK1‐KD forms under study were solvated using periodic dodecahedron boxes of TIP3P water molecules extending 10 Å from protein atoms and counter‐ions were used to neutralize each solvated system. Periodicity was applied to minimize edge effects. The solvated systems were optimized by steepest descent energy minimization and equilibrated by restrained MD simulations with harmonically restrained positions for protein (and peptide) atoms, in order to allow equilibration of the solvent, in two steps: 500 ps under NVT conditions followed by 500 ps in the NPT ensemble, with temperature kept at 300 K (separate coupling of protein and non‐protein atoms) and pressure at 1 bar. The optimization phase was followed by unrestrained, long, microsecond‐scale, MD simulations at 300 K in the NPT ensemble (production runs). Prior to the 300 K simulations, much shorter preparatory MD runs (and their corresponding equilibration steps) were carried out at 150 K, in order to better prepare the systems. The AMBER99SB‐ILDN force field that produces more reliable MD results [12], the PME method [29] for the treatment of the long‐range electrostatic interactions and a cut‐off of 8 Å for nonbonded interactions, were used in all simulations.

The MD simulations were carried out on a local 64‐core Dell PowerEdge R815 server.

### 2.6 Analysis of the MD Trajectories

Various types of analysis of the MD trajectories, namely, calculations of root‐mean‐square deviation (RMSD) of atomic positions from their initial positions and of root‐mean‐square atomic fluctuations (RMSF), analysis of geometric parameters such as atomic distances and dihedral angles, conformation clustering to obtain representative MD‐snapshots (in the last 500 ns of each MD trajectory) and principal component (PC) analyses to identify the dominant modes of atomic movements during the MD simulations, were carried out using GROMACS (details on the definition of such metrics can be found in [30]). The PC‐analysis, in particular, was performed in the last 500 ns of each MD‐trajectory and the movements of Cα‐ and sidechain‐atoms (a representative atom) were treated separately. Components PC1 to PC100 obtained from the independent PC‐analyses were saved as new trajectories (PC‐trajectories) and were used in subsequent dynamic allostery‐based community map analysis.

More specifically, community map analysis of the PC‐trajectories was performed using the Bio3D software [31, 32]. Contact maps produced using an atom–atom distance cut‐off of ≤ 10 Å for at least 75% of the corresponding simulation time on residues with correlated atomic movements (correlation coefficients, |*C*_*ij*_| > 0.5; obtained by cross‐correlation analysis of the corresponding PC‐trajectories) and the Girvan–Newman algorithm, as implemented in Bio3D, were employed for this purpose. The Girvan–Newman method (details in [33]) is a graph‐based network approach that offers an effective way for describing allosteric interactions, as it provides a quantitative estimate of allosteric coupling based on the edge‐betweenness measure from graph‐theory. Namely, the node‐betweenness centrality index of an individual amino acids reflects the influence of this residue in communication (modularity) and derives from the sum of shortest paths connecting other residue pairs that pass through it during the analyzed simulation time‐range. The results are then illustrated as 2D network‐graphs with nodes representing communities of residues (residues sharing high modularity values) and edges representing the strength of allosteric coupling between communities. Two independent community analyses; namely, of the Cα and side chain PC‐trajectories, were carried out for each one of the SRPK1 forms simulated in this study (four analyses in total).

The PyMOL Molecular Graphics System [34] and the VMD program [35] were used for model illustrations.

### 2.7 Cancer Mutations Search

Cancer‐associated mutations were obtained by screening the cBio Cancer Genomics portal [36] (at https://www.cbioportal.org) using the SRPK1 gene as query. This open access resource allows exploration of multidimensional cancer genomics data sets providing access to data from a large number of tumor samples from several studies. We run our query over 190 606 samples/181 877 patients from 382 studies (as to July 2023) and detected 509 mutations in the queried gene including 142 duplicate mutations in patients with multiple samples.

## 3 Results and Discussion

### 3.1 PIM‐1L Is Not a Substrate of SRPK1

**FIGURE 2:**
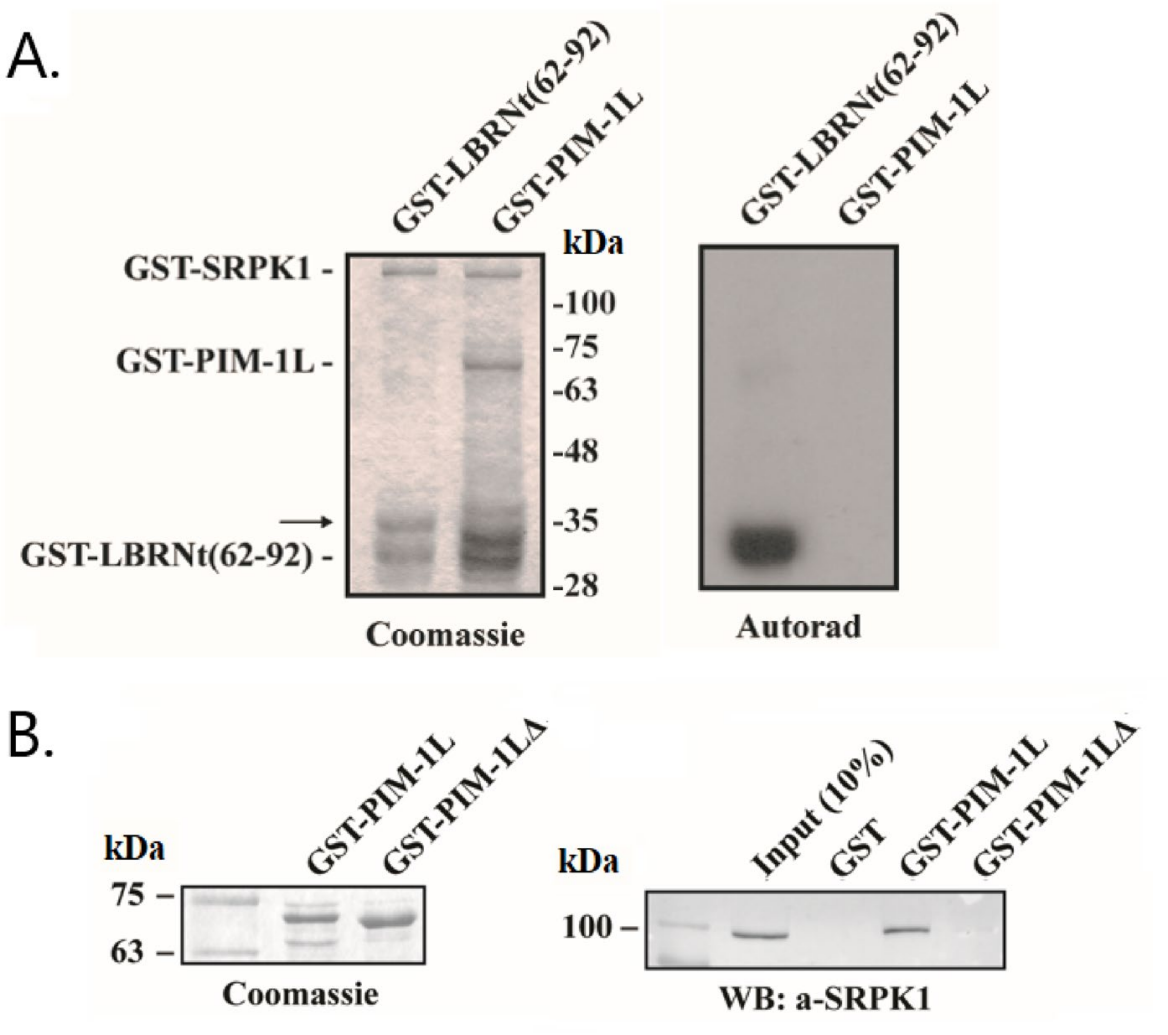
PIM‐1L interacts with but is not phosphorylated by SRPK1. (A) Phosphorylation of GST‐LBRNt(62‐92) and GST‐PIM‐1L (the full‐length fusion protein migrates with an apparent molecular mass of approximately 72 kDa; the lower bands represent degradation products) by GST‐SRPK1. The band indicated by the arrow represents a GST‐SRPK1 degradation product. Coomassie blue staining (*left panel*); auto‐radiography (*right panel*). Numbers on the right indicate molecular masses (in kDa). (B) *Left panel*, SDS‐PAGE analysis and Coomassie Blue staining of recombinant GST‐PIM‐1L and GST‐PIM‐1LΔ. Only the relevant part of the Coomassie stained gel is shown. *Right panel*, GST, GST‐PIM‐1L and GST‐PIM‐1LΔ immobilized on glutathione‐Sepharose beads were incubated with HeLa cell extracts. The sediments were harvested, washed three times with cell extraction buffer and analyzed by SDS‐PAGE. Bound SRPK1 was detected by Western blotting using the anti‐SRPK1 monoclonal antibody. A standard amount of cell extract, one‐tenth of which is shown, was used in each pull‐down assay. Numbers on the left indicate molecular masses (in kDa). This panel shows that the SR/SH‐rich domain of PIM‐1L is required for its interaction with SRPK1.

Given that the additional N‐terminal sequence of PIM‐1L contains a region rich in SR/RS and HS/SH dipeptides (Figure 1, amino‐acid stretch in red) we asked whether PIM‐1L could be phosphorylated by SRPK1. To this purpose, we initially performed in vitro phosphorylation assays using bacterially produced GST‐SRPK1 and GST‐PIM‐1L as substrate. As shown in Figure 2A and as expected, SRPK1 efficiently phosphorylated GST‐LBRNt(62‐92), a well‐characterized SRPK1 substrate [25]. On the contrary, it failed to modify GST‐PIM‐1L (Figure 2A), indicating that PIM‐1L is not an SRPK1 substrate.

### 3.2 PIM‐1L Binds to SRPK1 In Vitro and the SR/SH‐Rich Domain Is Essential for This Interaction

Despite the fact that PIM‐1L was not phosphorylated by SRPK1, we then tested the possibility whether these two proteins might interact with each other. To this end, GST‐PIM‐1L or GST immobilized on glutathione‐Sepharose beads were incubated with HeLa cell extracts. The beads were then harvested, washed extensively with PBST and bound proteins were analyzed by SDS‐PAGE and immunoblotting using the anti‐SRPK1 monoclonal antibody. As shown in Figure 2B, SRPK1 was able to associate with GST‐PIM‐1L but not with GST alone (Figure 2B, *Right panel*), indicating that PIM‐1L binds to SRPK1.

Having shown that PIM‐1L interacts with SRPK1, we next sought to define the protein segment in PIM‐1L mediating this interaction. To this purpose, we generated a GST‐PIM‐1L deletion construct, termed GST‐PIM‐1LΔ, lacking the SR/SH‐rich domain (deletion of residues 43–61 in red in Figure 1), and confirmed its correct protein expression (Figure 2B; *Left panel, GST‐PIM‐1LΔ lane*). Pull‐down assays were then used to test the ability of the GST‐PIM‐1LΔ construct to interact with SRPK1. As shown in Figure 2B (*Right panel*), and as opposed to the full‐length GST‐PIM‐1L protein, removal of the above sequence completely abolished binding (Figure 2B, *GST‐PIM‐1LΔ lane*), indicating that the SR/SH‐rich domain of PIM‐1L is essential for the PIM‐1L/SRPK1 interaction.

### 3.3 PIM‐1L Inhibits SRPK1 Activity In Vitro

**FIGURE 3:**
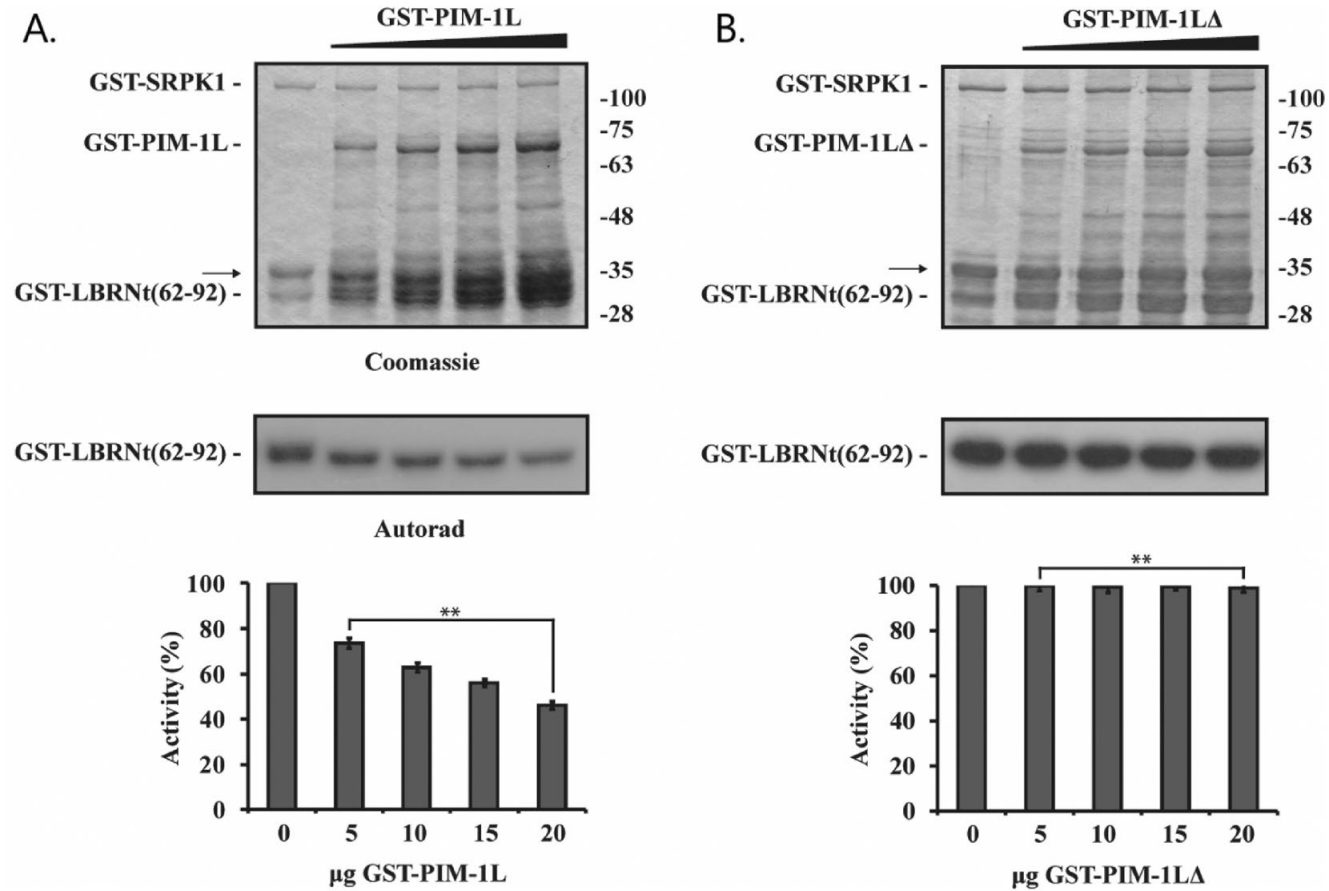
The SR/SH‐rich domain of PIM‐1L inhibits SRPK1 activity. GST‐SRPK1 kinase was incubated with GST–LBRNt(62‐92) and [γ‐32P]ATP in the presence of increasing amounts of GST‐PIM‐1L (A) or GST‐PIM‐1LΔ (B). The samples were analyzed by SDS‐PAGE, Coomassie blue stained (*upper panel*) and autoradiographed (*middle panel*, only the respective part of the gels is shown). The arrow as in Figure 2A. Phosphorylated bands were excised from the dry gel and kinase activity was estimated by scintillation counting (*lower panel*). Data represent the means ± SE of two independent experiments. *p* Values: *p* = 0.00652 (for GST‐PIM‐1L), *p* = 0.008053 (for GST‐PIM‐1LΔ).

To determine whether the interaction of PIM‐1L with SRPK1 had any effect on the kinase activity of SRPK1, we performed in vitro phosphorylation assays using as substrate bacterially produced GST‐LBRNt(62‐92), in the presence of increasing amounts of GST‐PIM‐1L. As shown in Figure 3, GST‐PIM‐1L inhibited the phosphorylation of GST‐LBRNt(62‐92) in a dose‐responsive manner (Figure 3A). Since the interaction of SRPK1 with PIM‐1L requires the PIM‐1L SR/SH‐rich domain (as shown above; Figure 2B, *Right panel*) we then asked whether the same protein segment was responsible for the observed inhibition of SRPK1 activity. To this end, we repeated the in vitro phosphorylation assays in the presence of increasing amounts of the GST‐PIM‐1LΔ construct. As shown in Figure 3B, deletion of its SR/SH‐rich region completely abolished the inhibitory activity of PIM‐1L.

Combined our observations so far, point to the SR/SH‐region as the binding domain mediating the SRPK1/PIM‐1L interaction and support the idea that this binding is responsible for SRPK1 inactivation.

We subsequently sought to investigate the atomic details of this interaction using in silico molecular modeling techniques.

### 3.4 Molecular Modeling Combined With MD Simulations Suggests That the PIM‐1L RS/SH Domain Can Mimic Docking‐Motif/Docking‐Groove Interactions With SRPK1

As already mentioned, SRPK1 uses its acidic docking groove (formed of the characteristic MAP kinase insert, the loop between helices αF and αG and helix αG) to bind basic peptides in its substrates and/or inhibitors that comply with a consensus sequence motif (docking motif) bearing arginine residues at motif positions 1, 3, and 7 (see e.g., [9, 11, 12, 15] and Figure S1A).

To explore the possibility whether the PIM‐1L SR/SH‐rich domain binds to SRPK1 in the same manner, we modeled into the SRPK1 docking groove a 9‐mer peptide located at the N‐terminal end of the PIM‐1L SR/SH‐rich domain that closely resembles to the canonical docking motif; namely, the amino‐acid stretch: 42‐FRASRSHSR‐50 (Figure S1B). The known crystal structure of a known SRPK1/substrate complex shown in Figure S1A (PDB ID code: 3BEG [11]) was used as template for this purpose (Section 2). It is of note that the modeled PIM‐1L peptide conforms to the consensus docking motif with the exception of the arginine residue at motif position 1, which is moved to position −1 in the initial 3D‐model of the complex simulated here (Figure S1B). We subsequently tested the validity of the model using a long (microsecond‐scale) all‐atom MD simulation (in explicit water). An extra 1 μs‐long MD simulation was performed in parallel on the SRPK1 APO‐form, for comparison.

The APO‐form simulation had converged after 500 ns of the 1 μs MD trajectory, whereas that of the complex (especially the PIM‐1L peptide part) required a longer simulation to converge (1.5 μs), as assessed by monitoring the RMSD of the Cα atoms from their starting position along the corresponding MD trajectory (Figure S2). The last 500 ns of each MD trajectory were thus used for subsequent analyses of the MD trajectories and representative snapshots of both simulated systems in this MD time‐range, were subsequently used to illustrate the MD results.

**FIGURE 4:**
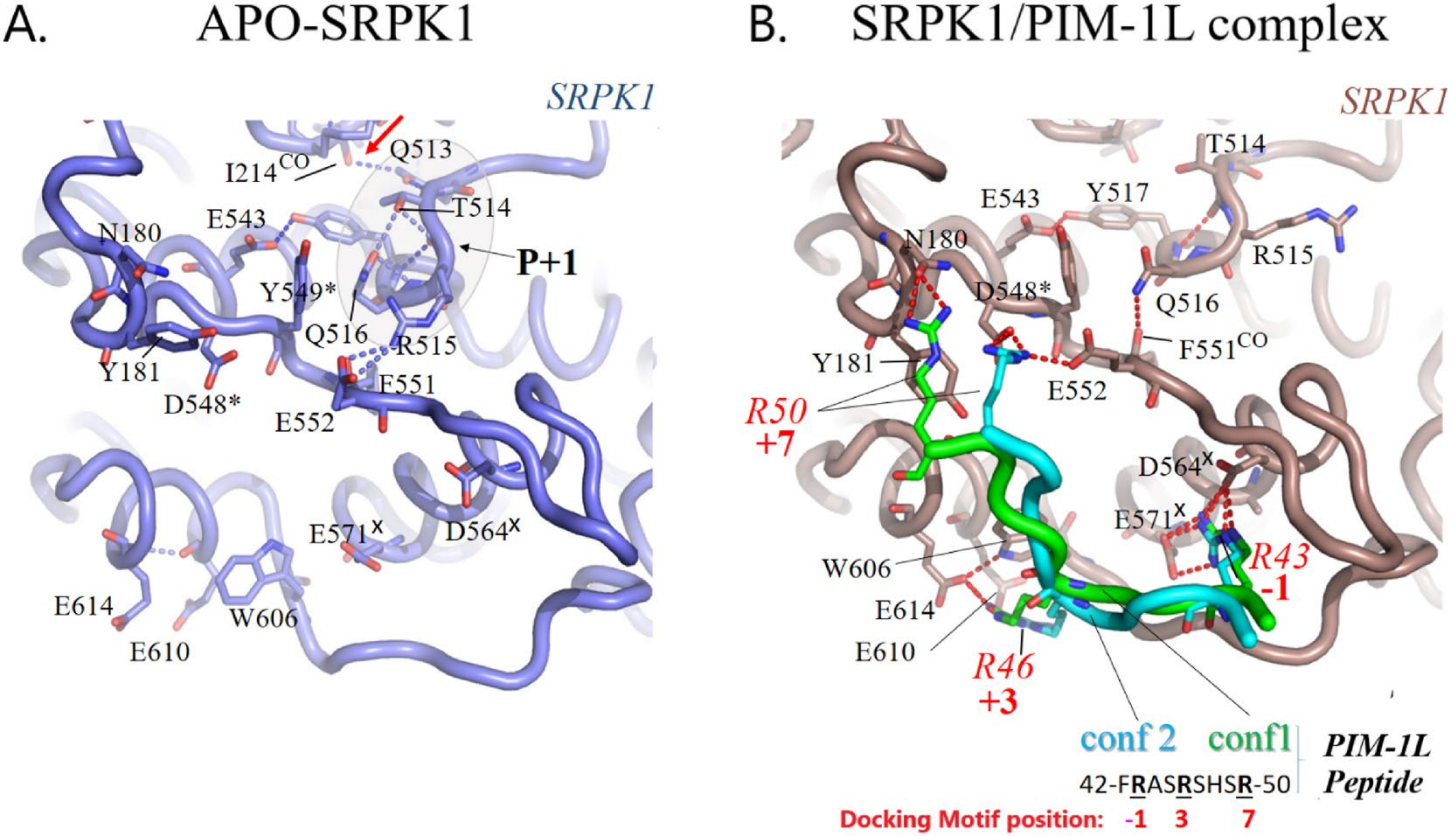
Atomic details of the interactions of SRPK1 with the PIM‐1L peptide simulated in this study. Ribbon representations of representative MD snapshots focused on the SRPK1 docking groove in its (A) APO and (B) PIM‐1L peptide‐bound forms, as obtained by cluster analysis of the last 500 ns of the corresponding MD trajectories. Two alternative conformations of the PIM‐1L peptide, are shown in green and cyan. Important residues discussed in the text are shown in sticks and labeled. Arginine residues of the PIM‐1L peptide, along with their corresponding docking‐motif positions, are red labeled (compare with Figure S1). Hydrogen bond distances are depicted as red dashed lines. Asterisks and x's indicate SRPK1 amino acids mutated in cancer: Missense or splice‐site mutations, respectively [36]. Rendering was made using PyMOL [34]. This figure demonstrates that the simulated PIM‐1L peptide can mimic docking‐motif/docking‐groove interactions seen in other SRPK1 complexes, suggesting that the PIM‐1L SR/SH‐rich domain may serve as a pseudo‐docking motif for SRPK1.

As shown in Figure 4B, the simulated PIM‐1L peptide interacts with SRPK1 by mainly charge complementarity involving ionic interactions between its arginine residues and known important acidic residues of the SRPK1 docking groove, such as D564, E571, E552, and D548, as observed in other known docking‐motif/docking‐groove SRPK1 interactions [9, 11, 12]. More specifically and interestingly enough, the side chain of the PIM‐1L arginine at motif position −1 (R43) flips inwards, toward the kinase, and interacts with the side chains of D564 and E571 (at helix αG), thus mimicking arginine residues at motif position 1 of canonical docking‐motifs (compare Figure 4B with Figure S1 at motif positions 1/−1). These interactions occur very early and remain stable during the entire MD trajectory of the simulated complex, as indicated by monitoring the corresponding side chain distances along the 1.5 μs MD trajectory and their narrow distributions at close distances (< 5 Å) (Figures S3A and S3B respectively, *upper panel*). Interestingly, a search of the cBioPortal for Cancer Genomics [36] revealed that amino acids D564 and E571 correspond to cancer‐related sites of the *SRPK1* gene, in support of our MD results reported here and in line with the importance of these residues in other docking‐motif/docking‐groove interactions (see e.g., [11, 12]).

The side chain of R46 at motif position 3, on the other hand, flips in the opposite direction compared with arginine residues at canonical docking motif position 3 (compare motif position 3 in Figure 4B and Figure S1A) and interacts with the SRPK1 docking groove through ionic interactions with the glutamate residues, E610 and E614 and a cation‐π interaction with tryptophan W606 (Figure 4B). All these PIM‐1L R46‐mediated interactions occur after approximately 600 ns of the MD trajectory of the complex and remain stable during the rest of simulation time (Figure S3, *middle panel*; note for example, the strong interaction with W606). The above‐mentioned SRPK1 residues are part of its MAP kinase‐like insert (in blue in Figure S1A). Tryptophan W606 in particular, has been reported to be the only amino acid that changes rotamer upon binding to docking motifs, as compared to the apo form of the enzyme, with a stabilizing role for arginine side chains at docking motif position 3 [9], although in a different manner compared to the R46/W606 stacking interaction observed in the SRPK1/PIM‐1L peptide complex of this study. Indeed, the W606 side chain exhibited higher dynamics in the APO simulation (two‐peak distribution of its *χ* ^2^ dihedral angle) as opposed to a single side chain conformation observed in the peptide‐bound simulation (Figure S4, *χ* ^2^ W606), in line with its proposed docking‐motif stabilizing role. In fact, W606 is one of the SRPK1 hydrophobic amino acids that exhibited altered side chain dynamics in the APO simulation, compared to the peptide‐bound case (Figure S4).

Finally, the PIM‐1L R50 at motif position 7, is more flexible but adopts conformations quite similar to those seen in canonical docking motif positions 7 (compare motif position 7 in Figure 4B and Figure S1A); namely, it interacts with side chains of residues E552 and D548 from the αF/αG loop (Figure 4B; see also Figure S3, *lower panel*). Similar flexibility of this motif position was also observed in the MD simulation of an LBR docking peptide in complex with SRPK1 [12]. The observation that D548 is replaced by an arginine residue in other MAP kinases (data not shown) and that it corresponds to a cancer‐related mutation site [36], further validate our MD results. It is also noteworthy that in the APO simulation, the side chain of E522 points away from the docking groove and is instead H‐bonded with an arginine of the substrate binding, P+1 loop; namely, R515 (Figure 4A) that corresponds to a substrate specificity determinant of Ser/Thr‐specific kinases (determinant 16 in [37]).

Taken together our MD results so far, strongly suggest that the SR/SH‐rich domain of PIM‐1L can target the SRPK1 docking groove and may act as a pseudo‐docking motif that mimics docking‐motif/docking‐groove interactions seen with other SRPK1 partners (substrates and/or inhibitors).

**FIGURE 5:**
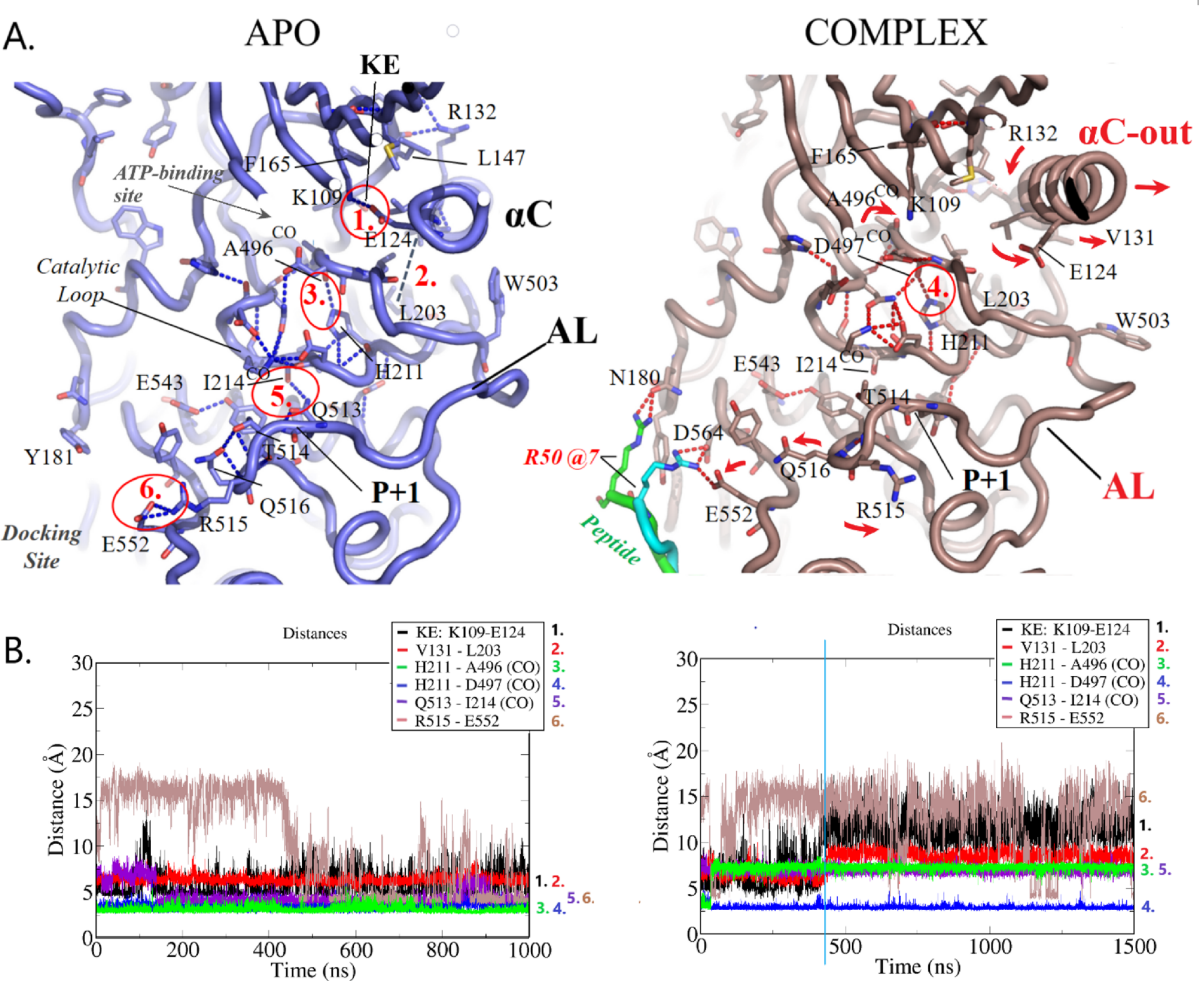
Comparison of intra‐molecular interactions in the vicinity of the SRPK1 active‐site. (A) Details of intra‐KD atomic interactions within SRPK1 in its two simulated forms, as observed in the corresponding MD snapshots (as in Figure 4). (B) Monitoring of important interatomic distances, encircled and numbered in red in panel A, along the corresponding MD trajectories. Red arrows indicate relative movements in the complex compared with the APO case. Binding of the PIM‐1L peptide is accompanied with disruption of various interactions including breakage of the KE salt‐bridge and an αC‐OUT conformation, indicative of inactive kinases.

We then sought to examine the effects of this binding outside the docking groove, and around the active site, in particular. Details of intra‐KD interactions in the two simulated SRPK1 forms focused on the surroundings of the catalytic center are shown in Figure 5.

### 3.5 The MD Results Suggest That Binding of the PIM‐1L RS/SH Peptide Induces Inactive Conformations of SRPK1

One hallmark of active kinases is a salt‐bridge formed between a conserved ATP‐binding lysine residue located in the N‐lobe β3 strand (K109 in SRPK1) and a glutamate residue of the critical, regulatory αC helix (E124). Breakage of the conserved KE salt‐bridge and a swing‐OUT conformation of αC, characterize inactive protein kinases [38].

As shown in Figure 5A, binding of the PIM‐1L peptide (*right panel*) is accompanied with conformational changes (indicated with red arrows) involving several intramolecular interactions observed in the simulated SRPK1 APO form (*left panel*). These changes are better illustrated by comparative monitoring of corresponding residue distances along the MD trajectories shown in Figure 5B and Figure S5. More specifically, a network of polar interactions (encircled in Figure 5A, *left panel*) that connect the docking site (at E552) and the substrate‐binding P+1 loop, with the catalytic center (through Q513) and the ATP‐binding region (KE‐site), although they remain stable during the APO‐SRPK1 simulation (Figure 5B, *left panel*), are highly disrupted in the simulated complex (distances >> 6 Å in Figure 5B; see also Figure S5). Interestingly, these disrupted interactions include breakage of the KE: K109‐E124 salt‐bridge after ~500 ns of the MD trajectory in the case of the complex (distance 1, >> 10 Å in Figure 5B, *right panel*; see also Figure S5a). Disruption of the KE salt‐bridge is accompanied with the αC‐OUT conformation seen in inactive kinases, as indicated by a much greater β3‐αC distance in the complex compared to the APO simulation (Figure S5b), suggestive of an inactive SRPK1 form in the presence of the docking‐groove‐bound PIM‐1L peptide.

An additional conformational change includes a backbone flip of A496, the amino‐acid immediately preceding the DLG sequence that corresponds to the conserved DFG‐motif in protein kinases. This flip (indicated by an arrow at A496 in Figure 5B), as reflected by the disruption of the H‐bond between the A496 backbone oxygen and the side chain ring of the conserved HxD‐histidine, H211 (distance 3 in Figure 5) in the complex, occurs very early and remains so during the rest of the corresponding MD simulation time (Figure 5B, *right panel*; and Figure S5d). Remarkably, such a peptide backbone flip was observed in a simulation of an inactive variant of PKA kinase, as suggested by a similar profile change of the conformational *ψ* angle of the corresponding amino acid (compare curve in red in Figure S4e of this study, with Thr183psi in Fig. S6 in [39]). This observation adds to the idea of an inactive SRPK1 form in the presence of the PIM‐1L peptide and further supports the notion that the nature of the xDFG‐residue is a determinant of inactive conformations of MAP kinases [40].

Another worth mentioning interaction that is affected upon PIM‐1L peptide binding involves a hydrophobic interaction between residues L203 and V131 of the SRPK1 inner‐hydrophobic core (distance 2 in Figure 5). This connects helices αC and αE and stabilizes the αC‐IN conformation in the APO SRPK1 simulated here (Figure 5A, *left panel*). Indeed, this van‐der‐Waals interaction (distance < 6 Å) is preserved in the APO simulation, whereas it is disrupted (distance > 8 Å, after 500 ns) in the case of the complex (compare distance 2 in Figure 5B; see also Figure S5c). In addition, as observed in the APO simulation, the bulky hydrophobic residue at the activation loop (AL), W503 (Figure 5A, *left panel*), may also contribute to stabilize the αC‐IN conformation and to connect αC with AL, an interaction that is also indicative of active protein kinases [41]. Notably, W503 adopts a completely different side chain rotamer in the PIM‐1L peptide‐bound SRPK1 (Figure 5A, *right panel* and Figure S4c,d) and is in fact one of the SRPK1 hydrophobic amino acids that exhibit altered side chain dynamics upon PIM‐1L peptide binding (Figure S4). Indeed, apart from W503, altered dynamics were also observed for various side chains of the inner‐hydrophobic core of the simulated SRPK1 forms, including those corresponding to conserved hydrophobic R‐, C‐spine [42] and R‐shell forming amino acids as well as to dynamic, spine‐bridging side chains [43] (Figure S4). More specifically, spine‐bridging side chains (including the “gatekeeper”) exhibited increased dynamics in the APO form (multiple‐peak *χ* ^1^ angle distributions), as opposed to quenched (single, narrow peaks) side chain dynamics of these residues in the simulated complex (Figure S4j–l). Given that increased dynamics in the inner‐hydrophobic core, especially of spin‐bridging side chains, is essential for dynamics‐driven allosteric activation of protein kinases [43], this observation also adds to the idea of an inactivating role of the PIM‐1L peptide.

Finally, a capping, regulatory role for residue R132 at the C‐terminal end of helix αC, similar to that observed in other active kinases (see e.g., the role of a conserved tryptophan residue in Tyr‐kinases, in [44]), is suggested by our MD results of the unbound kinase (Figure 5A, *left panel*). More specifically, in the APO form simulated here, the R132 side chain is located at a hydrogen‐bonding distance from the backbone‐oxygen of residue L147, which corresponds to one of the regulatory R‐spine‐forming side chains. This way it appears to assist blocking of the entrance to the inner‐hydrophobic core near the ATP‐binding (at the KE‐site; Figure 5A, *left panel*). This interaction (at hydrogen bonding distance < 3.5 Å) persisted during the APO simulation, whereas it was disrupted in the complex (Figure S5k) and was replaced by a H‐bonding interaction with the backbone oxygen of V144, instead (Figure S5l). The later conformational change of R132 resulted in turn, in an uncapping of the inner‐hydrophobic core and a water accessible ATP‐binding site in the case of the complex (Figure 5A, *right panel*). Validating our MD results and in support of a regulatory role of R132, this SRPK1 amino acid is a cancer‐hotspot [36].

Taken together our MD results so far, combined with the kinase activity experiments of this study (Figure 3), suggest that binding of PIM‐1L, through its SR/SH‐rich domain, promotes inactive conformations of SRPK1.

### 3.6 Community Map Analysis of the MD Trajectories Shed Light on the Dynamic Profile of SRPK1 in Its APO Form and in Response to PIM‐1L Peptide Binding

As shown above, our MD results suggest altered intramolecular communications within SRPK1‐KD in response to PIM‐1L peptide binding. To further investigate this issue, we carried out a dynamic allosteric‐based community analysis of the MD trajectories. Allostery is an inherent property of all dynamic proteins that allows remote (at distant sites) regulation, which is essential for their function and holds a key role in all signaling processes (a review on the concept of allostery can be found in [45]). Understanding allostery is necessary to understand biological mechanisms both in physiological and pathological conditions. Community network analysis of long MD trajectories, one of the methods to approach allostery, has been widely used in the literature and by us, to elucidate allostery in other protein kinases [39, 46, 47]. One of the first works of this type [46], in particular, suggested that active EPKs share the same dynamic architecture. Namely, their KDs are divided into dynamic communities (transient residue clusters moving as semi‐rigid bodies) with distinct functions. In brief, *ComA* that is responsible for ATP‐binding; *ComB*, which regulates positioning of the regulatory αC‐helix and of the ATP γ‐phosphate for transfer; *ComC* involved in regulation of kinase activity and in the assembly of the conserved regulatory R‐spine (assembled upon activation; i.e., AL phosphorylation); *ComD* that contributes to catalysis and the assembly of the catalytic C‐spine (assembled upon ATP‐binding); *ComE*, which is proposed to serve C‐spine assembly stabilization; *ComF* that is involved in activation of EPKs (phosphorylated AL‐OUT configuration) and in substrate binding; *ComG*, which is involved in protein–protein interactions including substrate binding and *ComH*, which is proposed to serve as docking [46] and scaffolding [47] communities. Community variations depending on the functional state of the kinase or mutations [39, 46] as well as additional communities, characteristic of each kinase family, are expected. For example, in our previous work on the tyrosine kinase TYK2, this type of analysis allowed us to identify a specificity‐determining community [47]. Comparison of the dynamic profiles of the two SRPK1 forms simulated in this study, is therefore expected to shed light on allosteric effects of PIM‐1L peptide‐binding to SRPK1. We thus sought to first elucidate the dynamic architecture of each one of the SRPK1 forms simulated here.

To this end, we applied community network analysis of the PC‐trajectories (Section 2). Main‐chain and side chain atomic motions were treated separately and communities were defined mainly by the Cα‐based (MC) community analysis. The side chain‐based (SC) community analysis was used to define extra communities, where necessary, and to obtain information on the boundaries of communities; namely, on bridging residues that have Cα and side chain atoms allocated to different communities. Nomenclature and coloring of the identified communities was according to their structurally and functionally equivalent communities identified in other protein kinases, whereas extra communities were defined based on SRPK1 specific secondary structure elements they are built around.

#### 3.6.1 Dynamic Architecture of the SRPK1 APO Form

**FIGURE 6:**
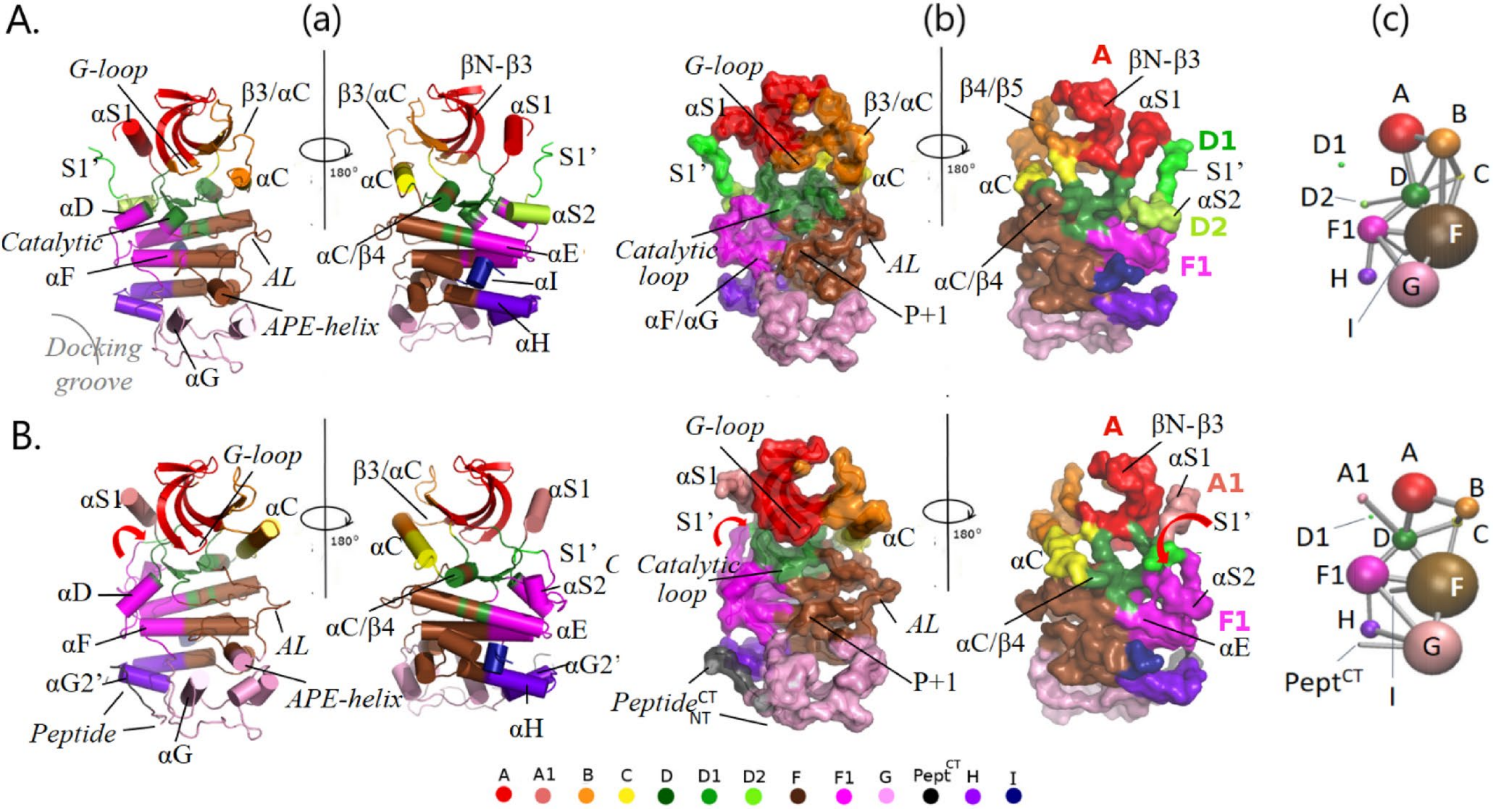
Community network analysis of the MD trajectories unravels the dynamic architecture of SRPK1. Main chain dynamic profiles of SRPK1‐KD in the APO form (A) and in complex with the PIM‐1L peptide (B), as obtained by Cα‐based (MC) community map analysis of the corresponding MD trajectories (last 500 ns). MC‐community residue‐members are mapped on the representative MD snapshots (as in Figures 4 and 5) and depicted as: (a) cylinder‐cartoons and (b) surface representations of their corresponding backbone atoms, (c) 2D‐graph illustrations of dynamics‐based allosteric MC‐community networks as obtained by Bio3D: Namely, nodes that represent communities are depicted as spheres with radii proportional to community size and edges that reflect allosteric coupling, are depicted as lines with widths corresponding to the strength of coupling between communities. Color‐index and nomenclature of communities are according to the color palette below the panel. Both front and back views of the kinase are shown, for clarity. Important secondary structure elements are labeled (compare with Figure S1). The red arrow indicates the relative movement of the S1′ element in the complexed SRPK1 compared with the apo enzyme.

The Cα‐based community map analysis of the APO SRPK1 MD trajectory detected 11 highly interconnected dynamic main chain communities, which are mapped on a representative APO MD snapshot and depicted using three types of illustration in Figure 6A (for community sizes refer to Figure S6A, *blue bars*). Among these, seven are structurally and therefore functionally equivalent to EPK communities described above; namely, Com‐A (red), ‐B (orange), ‐C (yellow), ‐D (dark green), ‐F (brown), ‐G (pink), and ‐H (violet). Interestingly, the C‐spine stabilizing *ComE* ^*PKA*^, is missing and merged with Com‐F1 (magenta), which in turn is structurally equivalent to the specificity determining community identified in TYK2 [47] (see also below). On the contrary, a separate Com‐I (dark blue) splits‐off from the protein–protein interaction community *ComH* ^*PKA*^.

Two extra communities, Com‐D1 and ‐D2, emerged from our analysis and appear to be SRPK1‐specific, as they are formed of residues of the non‐conserved spacer insert, which is characteristic of SRPKs (Figure S1A and [26]). More specifically, Com‐D1 comprises the backbone of the element of the spacer‐insert immediately preceding its αS2 helix (hereafter termed S1'), whereas Com‐D2 includes αS2 itself (Figure 6A‐a,b). Com‐D2 is a C‐lobe community and our analysis shows that it is allosterically coupled with the catalytic D community in the APO form (as indicated by a connection between spheres D and D2 in Figure 6A‐c). Com‐D, apart from regions related to catalysis, also includes part of the αC/β4 connecting region, strand β7 and the amino acid stretch immediately preceding the DLG‐motif (including the β8 strand and the DLG‐motif itself). Notably, these regions have been reported to move in block during N‐/C‐lobe relative movements in protein kinases (pivot block in [41]). Combined these findings suggest that Com‐D may also be considered as the pivot community in SRPK1 and that the spacer αS2 helix also contributes to relative movements of the lobes and to the optimization of the catalytic process. Com‐D1, on the other hand, is located in the interface between the lobes and therefore seems to serve inter‐lobe communication (Figure 6A,B and Figure S1A). This connection, however, appears to be highly dynamic and transient as indicated by the dynamic character of the S1' element (Figure S7) and lack of strong allosteric coupling involving Com‐D1 (lack of connecting lines of D1 sphere in Figure 6A‐c).

On the contrary, the spacer helix αS1 is much less dynamic (Figure S7) and is clearly part of the ATP‐binding community (Com‐A) together with a 4‐standed β‐sheet (βN to β3; in red in Figure 6A‐a,b). This observation indicates that αS1 contributes to the assembly of the N‐lobe β‐structure and to correct positioning of the ATP‐adenine, supporting the idea that the spacer helices serve stabilization of the SRPK1 N‐lobe conformation [26].

It is also worth mentioning that the other SRPK characteristic region, namely, the MAP kinase‐like insert (in blue in Figure S1A), which contributes to forming the docking groove as described earlier, is split in two communities in the APO simulation. Namely, Com‐G (along with the αG helix; substrate docking/binding community) and Com‐H (along with the αΗ helix; protein–protein interaction community, also contributes to docking‐groove formation). Interestingly, these communities are allosterically uncoupled in the APO SRPK1 simulation, as indicated by lack of linking between spheres G and H in Figure 6A‐c.

#### 3.6.2 APO Side Chain Communities: The Major Interconnecting Community Is Related to Substrate Specificity/Recognition

**FIGURE 7:**
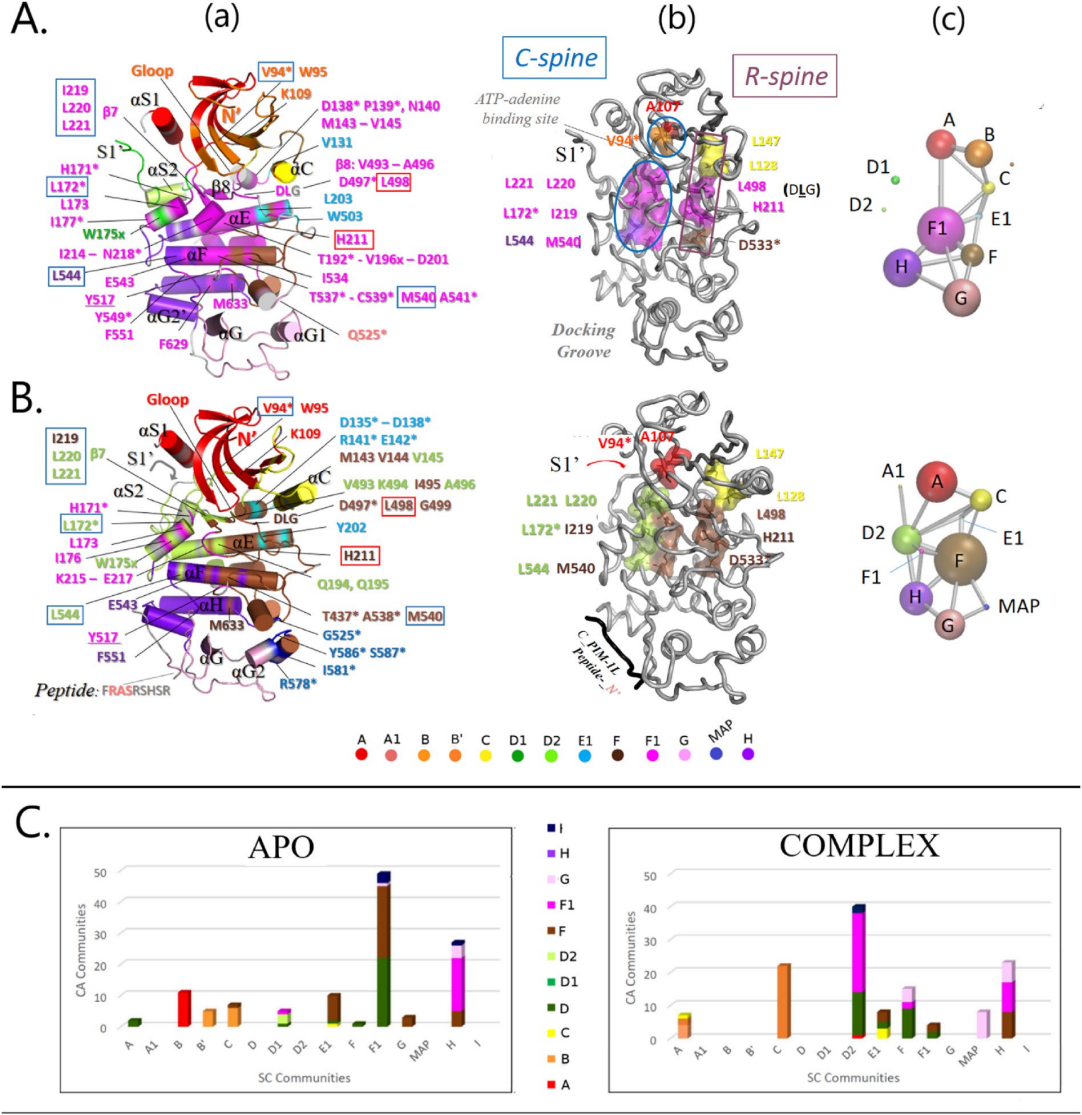
Side chain dynamic profiles reveal dramatically altered allosteric communications within SRPK1 in response to PIM‐1L peptide binding. Results obtained by the side chain‐based community network analysis of the MD trajectories of (A) APO‐ and (B) PIM‐1L bound SRPK1. (a) SC‐community based cylinder‐cartoon representations and (b) surface representation of SRPK1 residues corresponding to conserved spine‐forming side chains, mapped on the corresponding MD‐snapshots. (c) 2D‐graph illustrations of the allosteric side chain community networks. Coloring is according to the corresponding SC‐communities (horizontal color palette). Red‐ and blue‐boxed labels in *panel* (*a*) and boxes in *panel* (*b*), indicate R‐ and C‐spine forming residues, respectively. The backbone of SRPK1 and of the PIM‐1L peptide in *panel* (*b*) are depicted as tube‐cartoons. Asterisks and x's, as in Figure 4. As expected, due to the absence of ATP, the C‐spine is incomplete in both cases. On the contrary, and despite the absence of AL‐phosphorylation, the R‐spine is assembled in the simulated APO SRPK1, whereas it is broken in the case of the complex. (C) Bar plots of community‐bridging side chains in the two simulated SRPK1 forms; The term “bridging” is used for residues with Cα and side chain atoms allocated in different communities. The x‐axis corresponds to SC‐communities, whereas the *y*‐axis shows the number of bridged MC/SC community pairs with bars colored according to their corresponding MC‐communities (vertical color index).

The SC‐based community analysis of the MD trajectory (Figure 7A and Figure S8), on the other hand, identified two extra solely side chain communities in the APO form. Namely, Com‐B'^SC^ and Com‐E1^SC^ (with 5 and 10 members, respectively; Figure S6B, *blue bars*). Com‐B'^SC^ includes side chains of the β3/αC connecting loop, whereas Com‐E1^SC^ is formed of hydrophobic side chains including those of V131, L203, and W503 (Figure 7A‐a and Figure S8f). As described earlier, our MD results suggest that these side chains serve stabilization of the active αC‐IN conformation and its dynamic connection to the AL in the APO case. Indeed, as better illustrated in Figure 7C (*left panel*), Com‐E1^SC^ bridges the regulatory community C with the activation community F and the catalytic/pivot D community (Figure 7C, *left panel*; yellow, brown and dark green stripes, in bar at “E1”). It also mediates allosteric coupling of Com‐C^SC^ with ‐F^SC^ and ‐F1^SC^ (Figure 7A‐c). It can be therefore concluded that E1^SC^ contributes to dynamic support of the R‐spine assembly.

The most populated APO SC‐community is Com‐F1^SC^ (Figure S6B and Figure S8d), which is structurally equivalent to the specificity‐determining community we previously identified in a tyrosine kinase, as already mentioned (Com‐FG in [47]). Indeed, and as shown in Figure 7A‐a, Com‐F1^SC^ includes the side chains of several specificity determining amino acids such as H171, L172, L173, E217, and E543 that correspond to determinants 6, 7, 8, 10, and 19 in Ser/Thr‐specific kinases [37]. More importantly, it is defined by the side chain of the AL‐residue (at P+1) Y517, which is characteristic of Ser/Thr‐specific kinases (vs. a tryptophan residue at this position in Tyr‐specific ones [48]) and has been reported as an allosteric hotspot (see e.g., role of Y204^PKA^ in [39]).

Notably, Com‐F1^SC^ is also the major bridging community in the APO form (Figure 7C, *Left panel*; bar at “F1”). As already mentioned, active EPKs are characterized by assembled spatial motifs, termed spines, that serve noncovalent connection between the kinase lobes, which is necessary for kinase activity. The regulatory R‐spine depends on the configuration of the AL and is conserved in different kinases, whereas the catalytic C‐spine is transiently assembled in the presence of ATP and is sensitive to the nature of the substrate [42]. Notably, five out of the eight C‐spine forming side chains (L172, L219‐L221, M540) are Com‐F1^SC^ members in the APO SRPK1 simulated here (Figure 7A‐b). This finding, combined with the replacement of ComE^PKA^ by Com‐F1 in SRPK1, is in line with the aforementioned notion that the C‐spine assembly is also substrate dependent.

Interestingly, the APO Com‐F1^SC^ also includes two R‐spine forming residues: H211 and L498, suggesting that the R‐spine assembly is also dependent on substrate specificity in the case of SRPK1. Moreover, our analysis shows that both these amino acids are bridging residues that serve dynamic connection of the specificity‐determining community with the activation (F^CA^) and the catalytic (D^CA^) communities, respectively. Notably, H211 corresponds to the HxD‐motif histidine that is however, replaced by a tyrosine residue in ~8% of EPKs, whereas L498 corresponds to the conserved DFG‐motif phenylalanine in other kinases and a leucine residue at this position appears in only ~7% of EPKs [49]. It has been reported that a leucine replacement for the DFG‐phenylalanine can be tolerated in other kinases: namely, such amino acid change was found to have no effect on the catalytic activity of PKA [49] and to result in a variant that populates predominantly the active conformation in Abl kinase [50]. Our Com‐F1^SC^ results, combined with the observation that L498^SRPK1^ dynamically bridges catalytic activity with substrate specificity/recognition (Com‐D^CA^/Com‐F1^SC^) suggest that the effect of a leucine residue at this position is at the dynamic level and related to the nature of the substrate in the case of SRPK1. To the best of our knowledge, such connection between the R‐spine assembly and substrate specificity/recognition has not been appreciated before. Moreover, as shown in Figure 7B‐b, apart from the observation that Com‐F1^SC^ is the most represented SC‐community in both spines, the R‐spine is assembled (dark red box) in the APO enzyme, despite the absence of AL‐phosphorylation in the template structure and our model. This finding is in agreement with literature data showing that the SRPK1‐KD is constitutively active [26].

Com‐F1^SC^ also comprises additional long hydrophobic side chains serving bridging of the spines in active EPKs [43], such as V144, V145, V196, I214, V493, I495 (Figure 7A‐a). The above findings are reflected in the allosteric profile in Figure 7Ac showing that F1^SC^ remains allosterically coupled with almost all, both N‐lobe and C‐lobe SC‐communities in the APO simulation, with the exception, however, of Com‐B and the spacer communities, D1 and D2. Given that Com‐B^APO^ includes the G‐loop in the APO case (Figures 6A‐b and 7A‐a), the latter observation indicates that the G‐loop and the spacer elements S1′ and αS2 do not contribute to determining substrate specificity in SRPK1.

Combined, these findings in conjunction with the notion that bridging of the spines is necessary for dynamics‐driven allosteric activation [43], point to Com‐F1^SC^ as the spine bridging community and therefore, as a key player in securing specific allosteric activation of SRPK1. Interestingly, this side chain community is dramatically less populated and the R‐spine is broken in the complex (see below), suggesting that binding of the PIM‐1L peptide into the docking groove, interferes with dynamics‐driven allosteric SRPK1 activation. The dynamic architecture of SRPK1 in response to PIM‐1L peptide binding, is presented in details in the next section.

#### 3.6.3 Dynamic Architecture of the Complex: The Dynamic Profile of SRPK1 Is Severely Altered in Response to PIM‐1L Peptide Binding

The Cα‐based community map analysis of the corresponding MD trajectory identified 12 dynamic MC‐communities in the SRPK1/PIM‐1L peptide complex (Figure 6B) that differ from those of the APO form both in membership (for community sizes refer to Figure S6A) and connectivity. Among these, an extra N‐lobe community, Com‐A1, splits‐off from Com‐A^APO^ and comprises the spacer helix aS1 (Figure 6B‐a,b), which is now detached from the ATP‐binding βN‐β3 domain (compare αS1 in Figure 6A,B‐a,b). Interestingly, Com‐A1 and Com‐A are allosterically uncoupled (Figure 6B‐c), indicating that in contrast to the APO SRPK1, the spacer helix αS1 does not contribute to the stabilization of the N‐lobe β‐structure in the PIM‐1L peptide bound enzyme. On the contrary, two other communities: namely, Com‐D2^APO^ and Com‐F1^APO^, although separate and lacking allosteric connection in the APO form (Figure 6A‐c), are merged into one in the complex (Com‐F1^CPLX^; compare *right panels* of Figure 6A,B‐a,b). This merged community is defined as F1 (instead of D2) due to a larger overlap with the APO F1^CA^ community. Both the merging of D2 and F1 communities and the split of Com‐A, appear to result from a flip of the spacer element S1′ toward αS2, in the complex (compare S1′ positions in Figure 6A,B‐b). This S1′ flip is accompanied with a rigidification of the C‐terminal portion of the spacer (comprises S1′ and αS2) in the complex as opposed to the APO form (Figure S7).

An additional important difference between the two simulated SRPK1 forms is related to another N‐lobe community, Com‐B. More specifically, Com‐B^CPLX^ does not include the G‐loop, which is now part of Com‐A^CPLX^, instead (Figure 6B‐a,b). Notably, Com‐B remains dynamically coupled with the catalytic D and the activation F communities in the APO form, whereas these connections are abolished in the case of the complex (compare connections of sphere B in Figure 6c). Indeed, the G‐loop exhibits higher fluctuations in the APO case versus the complex (Figure S7). Combined these findings, in conjunction with the notion that dynamic G‐loops are indicative of active kinase forms [41], further support the idea of an inactivating effect of the PIM‐1L peptide. Moreover, the observation that Com‐B^APO^ is coupled with Com‐A^APO^ (Figure 6A‐c) suggests a dynamic communication between the G‐loop and αS1 in the apo enzyme, which is also abolished upon PIM‐1L peptide binding, as inferred by loss of coupling of the corresponding communities in the complex: namely, Com‐A1 (includes αS1^CPLX^) and Com‐A^CPLX^ (includes G‐loop^CPLX^), respectively (Figure 6B‐c).

The PIM‐1L peptide on the other hand, is split in two communities (Figure 6B‐b, *left panel*). Namely, its N‐terminal part (aa: 42‐FRAS‐45; motif positions −2 to +2) is incorporated into Com‐G, whereas its C‐terminus forms a separate community (pept^CT^), which however remains coupled with Com‐G^CPLX^ (Figure 6B‐c). In addition, binding of the peptide seems to contribute to the allosteric coupling between the docking‐groove‐forming communities, Com‐G^CPLX^ and Com‐H^CPLX^ (Figure 6B‐c), an allosteric connection that is lacking in the APO simulation (Figure 6A‐c). On the contrary, a dynamic coupling of Com‐F1 with Com‐I, observed in the APO case, is disrupted in the complex (Figure 6C).

#### 3.6.4 Side Chain Communities in the Complex: The Substrate‐Specificity‐Determining Community Is Underrepresented and Largely Replaced by a Spacer‐αS2‐Centered One

As illustrated in Figure 7 and Figure S6, the SC‐based community network analysis revealed even more dramatic effects of the PIM‐1L peptide binding on the dynamic architecture of SRPK1 side chains (see also Figure S8). For example, apart from the extra A1 community described above, an additional 8‐membered and solely SC‐community was detected in the complex; namely, MAP‐ins (Figure S6B). This community includes side chains of the αG1 and αG2 helices of the MAP kinase‐insert and of their connecting loop (in blue in Figure 7B‐a, see also Figure S1B). This community splits‐off from Com‐G^APO^ (Figure S8h) but remains coupled with it as well as with the activation community F during the simulation of the complex (Figure 7B‐c). It is noteworthy that the majority of the Com‐MAP forming side chains correspond to cancer‐related mutations of the *SRPK1* gene, as revealed by screening the cBioPortal for Cancer Genomics [36] and this implies an important role of this domain in allosteric SRPK1 activation. Indeed, this site is part of a docking site for regulatory proteins in other kinases (docking site A in [42]).

Additionally, the SC‐based community analysis revealed significant differences even between common SC‐communities of the two simulated SRPK1 forms (Figure 7 and Figure S8). These differences are more pronounced in the case of communities F1^SC^ and B^SC^ in the APO, and F^SC^ and D2^SC^ in the peptide‐bound forms, respectively (Figure S6B). More specifically, and as already mentioned, the most interconnecting APO SC‐community, Com‐F1^SC^, is much less populated in the complex (compare Com‐F1^SC^ in Figure 7, Figures S6B and S8d): it includes the side chains of only few specificity‐determining amino acids (four vs. six in the APO form: Y517, H171, L173, and E217), but lacks spine‐forming or spine‐bridging side chains (Figure 7B‐a). This finding along with the observation that in contrast to the APO case, Com‐F1^SC^ abolishes coupling with the N‐lobe communities Com‐A^SC^ and Com‐C^SC^ (Figure 7B‐b), indicates interference with substrate specificity‐related allosteric communications between the lobes.

More specifically, the major interconnected SC‐community in the complex is Com‐D2^SC^ (Figure 7B‐c,C, *right panel*), instead. Indeed, along with the αS2 helix, four out of 10 spine‐bridging F1^APO^ side chains (V145, L220, L221, V493) and five C‐spine‐forming ones (I219‐L221, L172, L544), are incorporated into Com‐D2^SC^ in the complex (Figure 7B‐a). However, Com‐D2^SC^ lacks R‐spine‐forming side chains, which in turn, are part of Com‐F^SC^ in the complex (see e.g., H211, L498 in Figure 7B‐a). Com‐F^SC^ is the most populated SC‐community in the complex (Figure S6B) and apart from the aforementioned R‐spine, also incorporates another 27 F1^APO^ side chains, including those of C‐spine‐forming and spine‐bridging amino acids (I214, I219, V144, I495, V496, M540) as well as a larger portion of the αF‐helix (Figure 7B‐a). Notably, Com‐F^CPLX^ is the largest among the SC‐communities of both SRPK1 forms (Figures S6B and S8) and the fact that it covers a larger portion of αF (instead of Com‐F1^SC^ in the APO form), is reminiscent of a similar finding reported in the literature: namely, αF was found to reside mainly in *ComF* in a simulated inactive PKA form [46]. Combined these observations, further support the idea of an inactive PIM‐1L peptide‐bound SRPK1.

The second APO SC‐community, which is highly populated in the APO form but is completely missing in the simulated complex is Com‐B^SC^ (Figure S6B). The APO Com‐B^SC^, apart from the G‐loop (which normally binds the ATP‐phosphates and also assists substrate binding in EPKs), also includes the side chain of the ATP‐binding lysine K109 of the KE salt‐bridge and the N‐lobe C‐spine residue, V94 (orange in Figure 7A‐a). On the contrary, these side chains belong to Com‐A^SC^ in the complex (red in Figure 7B‐a). In fact, the APO N‐lobe Com‐A^SC^ and Com‐B^SC^ are merged into one large community in this case (Com‐A^CPLX^, Figure S8a).

Taken together, the community network analysis results so far suggest that dynamic allosteric signaling necessary for kinase activity is disrupted in the presence of the PIM‐1L peptide. We thus sought to identify key residues that mediate allosteric signal transmission within each simulated form.

### 3.7 Identification of Amino Acids Serving Dynamics‐Aided Allosteric Signal Propagation Within SRPK1

#### 3.7.1 Main Chain‐Mediated Allosteric Communications

**FIGURE 8:**
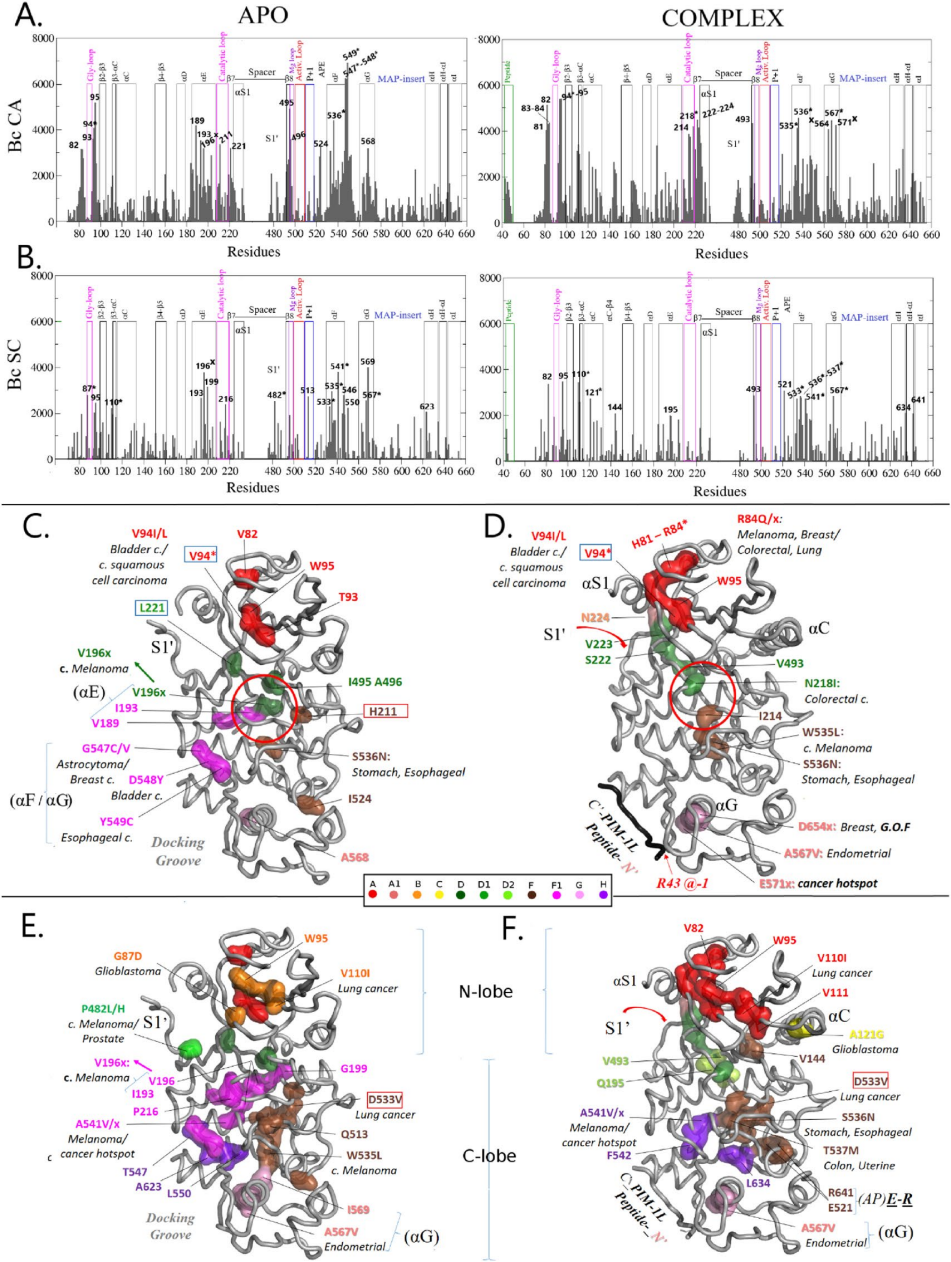
Identification of amino acids with a pivotal role in mediating allosteric signal propagation within SRPK1 in its APO and peptide‐bound forms. (A, B) Node‐betweenness centrality plots: Betweenness centrality values for (A) Cα atoms (Bc^CA^) and (B) representative side chain atoms (Bc^SC^), as obtained by Bio3D analysis of the corresponding MD trajectories are plotted along the primary structure. Residues characterized by significantly higher Bc^CA^ and/or Bc^SC^ values (top 5% of Bc values) suggesting a pivotal role in dynamics‐driven allosteric signal transmission within the simulated SRPK1 forms, are labeled. Secondary structure elements and functionally important regions and conserved motifs, are boxed and labeled (see also Figure S1A). (C–E) Community‐based illustrations: Strongly signaling main chains (*C, D panels*) and/or side chains (*E, F panels*), labeled in panels (A) and (B) respectively, are mapped on the corresponding MD‐snapshots and depicted as surfaces colored according to their MC and/or SC communities, respectively. Amino acid labeling is colored accordingly, for comparison purposes. Corresponding cancer‐associated missense and/or splice‐site mutations (marked with asterisks and in ‘x's in panels A and B) and information on related amino acid changes and cancer types, are indicated where applicable. Red circles in panels (C) and (D) denote the kinase active‐center. Boxed labels as in Figure 7 and arrow as in Figure 6.

To identify amino acids that contribute the most to allosteric communications, node‐betweenness centrality (Bc) values, as obtained, first, from main chain (Cα‐based) Bio3D analysis of the MD trajectories (see Section 2.6), were extracted and plotted along the amino acid sequence (Figure 8A). Residues characterized by significantly higher node‐betweenness centrality values (top 5% of Bc^CA^ values), indicating a pivotal role of their backbone in mediating allosteric signaling within each SRPK1 form, are labeled in Figure 8A. The main chain atoms of these residues were subsequently mapped on MC‐community‐based illustrations of the corresponding MD snapshots of the two SRPK1 forms and are shown in Figure 8C,D.

As shown in Figure 8A (*Left panel*), the strongest contribution to allosteric communications in the APO form, is that from the backbone at residues G547 to Y549 (Figure 8A, *left panel*). These amino acids are located in the αF/αG connecting loop (Figure 8A, *left panel*) and functionally belong to the substrate specificity‐determining community, Com‐F1^CA^ (Figure 8C). Other strong mediators of allosteric signal propagation include the main chains of the N‐lobe residues: V82 (β1), T93 and V94 (β2), the αE residues: V189, I193, and V196, the HxD histidine, H211 (β6; R‐spine), the C‐spine residue L221 (β7) and the pivot, I495‐A496 (immediately preceding the DLG‐motif) as well as the main chains of I524, S536, and A568 that are located in the “APE”‐, αF‐ and αG‐helices, respectively (Figure 8A, *Left panel*). This allosteric signaling serves therefore, dynamic connection of the N‐lobe β‐structure with the C‐lobe β‐ and α‐structures as well as with the substrate‐binding αF/αG region, reflecting long‐range dynamics‐driven allosteric signaling connecting the kinase lobes in the unbound SRPK1. This seems to be facilitated by increased dynamics of several of the aforementioned regions in the APO simulation versus the complex (see e.g., the higher fluctuations of the N‐terminal half of αE and of the αF/αG connecting loop in Figure S7).

In the case of the PIM‐1L peptide‐bound SRPK1, on the other hand, strong allosteric signaling lacks contribution from the αF/αG region or from the spine residues, H211 and L221, as shown in Figure 8A (*Right panel*). It also does not include the αE helix and its spine‐bridging, V196. Instead, it involves the backbone from the pivot‐block [41] around which helix αC swings out: namely, I214, N218, and V493 (Figure 8D) as well as of the αG helix at residues D564, A567, and E571 (Figure 8A, *Right panel*; and Figure 8D). Notably, the latter three residues are components of the acidic docking groove and their side chains are involved in binding the docking peptide arginine at motif position −1, as described earlier (Figure 4B).

#### 3.7.2 Side Chain‐Mediated Allosteric Signal Propagation Within SRPK1

It is well‐established that allosteric signal propagation is mainly mediated by side chains and especially dynamic hydrophobic ones, as already mentioned [43, 47]. We therefore, subsequently focused on the side chain‐based Bio‐3D analysis of the MD trajectories. For this purpose, side chains mediating strong allosteric signal transmission (with top 5% of Bc^SC^ values, labeled in Figure 8B), were also added to the corresponding MC‐community‐based illustrations and are shown in Figure 8E,F.

As shown in Figure 8B (*Left panel*) and better illustrated in Figure 8E, strong allosteric signaling in the APO simulation includes side chains from both the N‐lobe β‐structure (W95, V110 and the backbone oxygen of G87 from the G‐loop) and the C‐lobe α‐helices (the universally conserved D533 and W535 from αF; I193, V196, and G199^CO^ from αE and the αG residues, A567 and I569) as well the side chains of P216 from the catalytic loop, Q513 from the substrate‐binding P+1 portion of the AL, and of the spacer residue P482 (Figure 8E). Supporting their pivotal role, the majority of the corresponding amino acids (47%) are related to cancer‐causing mutations (Figure 8E) and several correspond to conserved ones with key roles in other active protein kinases.

For example, the dynamics of the equivalent of the αE spine‐bridging hydrophobic side chain of V196, has been proposed to serving coupling of substrate recognition with ATP binding and catalysis in EPKs (see e.g., the role of I950^PKA^ in [43]). In line with this notion, our analysis revealed that in the APO form, the N‐terminal portion of αE is more dynamic (Figure S7) and both the main chain and side chain of V196 are allosteric hotspots bridging the catalytic community, Com‐D^CA^ (Figure 8C, green arrow) with the specificity‐determining community, Com‐F1^SC^ (Figure 8E, magenta arrow). Further supporting this finding, a splice site mutation at position 196 results in cutaneous melanoma (Figure 8E). Noteworthy is the observation that replacement of three additional side chains among those with higher Bc^SC^ values in the APO form, also results in melanoma (Figure 8E). These include two side chains from the signal integrator, αF: namely, the universally conserved W535, which is a centerpiece of the conserved hydrophobic kinase core and A541, which corresponds to a long hydrophobic side chain in other kinases and serves anchoring of the αH helix [42] as well as the spacer residue P482 (Figure 8E).

Based on these observations, and apart from V196, P482, and W535, several additional long hydrophobic SRPK1 side chains emerged from our analysis as potential spine‐bridging in the active APO form: namely, W95, V110, I193, P216, L550, and I569 (Figure 8E). Interestingly, among these, only two N‐lobe side chains (W95 and V110) are shared with the PIM‐1L complexed form, whereas the remaining, C‐lobe ones, are lacking in the latter. On the contrary, short hydrophobic but strongly signaling side chains of the C‐lobe (A541 and A567) along with that of the universally conserved and R‐spine‐regulating aspartate residue, D533, were identified in both forms (Figure 8E,F). Noteworthy is the observation that alanine residues at positions 541 and 567 are unique to SRPKs and their replacement by the longer hydrophobic side chain of valine, also results in several types of cancer (Figure 8E,F).

### 3.8 Prediction of the Functional Role of the Identified Allosteric Signaling Mediators: Implications in Cancer

To further elaborate on the functional significance of the identified strongly signaling side chains in the light of our community map analysis results, residues with high Bc^SC^ values in each SRPK1 form, were classified according to their corresponding communities and in conjunction with predicted community‐based functions (see Section 3.6) and involvement in cancer, are collectively shown in Table 1.

**TABLE 1:** Collective listing of side chains mediating dynamic allosteric communications within APO and PIM‐1L‐bound SRPK1, identified in this study.

MC‐Community (predicted functional role)	SC‐Community (predicted functional role)	APO	COMPLEX	SSE	Literature	Mutations identified in cancer patients (*)	
						Protein change	Cancer type
**‐A:** ATP‐binding; dynamic assembly of the N‐lobe β‐structure, correct positioning of the ATP‐adenine ring	‐**A:** Dynamic assembly of the N‐lobe β‐structure		**V82**	β1			
			**W95**	β2			
			V110*	β3		V110I	Lung
			**V111**	β3			
	**‐B:** Contributes to dynamic N‐lobe β‐structure, binding of ATP‐phosphates, αC‐IN positioning	G87*		G‐loop	(a)	G87D	Glioblastoma
		**W95**		β2			
**‐B:** Dynamic αC positioning, ATP‐phosphates binding	**‐B:** αC‐IN positioning, ATP‐phosphates binding	V110*		β3		V110I	Lung
	**‐C:** Regulatory		A121*	aC		A121G	Glioblastoma
**‐D:** Catalytic; Pivot community (putative)	**‐D**2: C‐lobe Spacer community, Pivot		**Q195**	aE	(a)		
			**V493**	β8	(a), (c), (f)		
	**‐F1:** Specificity determining, Spine‐bridging, Pivot	**V196** ^x^		aE	(c), (d), (e)	V196 x	c. Melanoma
		**G199**		aE	(a), (b), (f)		
	**‐F:** Activation (AL‐OUT conformations)		**V144**	αC‐β4	(d), (e), (f)		
**‐D2:** Spacer C‐lobe community	**‐D1:** Spacer N‐lobe community	P482*		Spacer, S1’	(g)	P482 H/L	Prostate/c. Melanoma
**‐F:** Activation, Substrate binding	**‐F:** Activation, Substrate binding	**Q513**		P+1			
			**E521**	(AP) **E**	(h)		
		D533*	D533*	aF	(a), (i)	D533V	Lung
		W535*		aF		W535L	c. Melanoma
			S536*	aF	(a)	S536N	Stomach, Esophageal
			T537*	aF		T537M	Colon, Uterine
			**R641**	(AP)E‐ **R**	(h)		
	**‐H:** p/p interactions		**L634**	aH			
**‐F1:** Substrate recognition/binding, Specificity determining	**‐F1:** Specificity determining, Spine‐bridging	**I193**		aE			
		**P216**		Catalytic Loop			
		A541*		aF		A541V/ x	c. Melanoma/cancer HOTSPOT
	**‐H:** p/p interactions: Contributes to docking groove formation		A541*	aF			
			**F542**	aF			
		**T546**		aF	(b)		
		**L550**		aF/αG	(j)		
**‐G:** Assists substrate binding, Docking groove formation	**‐G:** Docking groove formation, Docking motif interactions	A567*	A567*	αG		A567V	Endometrial
		**I569**		αG			
**‐H**; p/p interactions	**‐H:** p/p interactions	**A623**		aH	(a)		

*Note*: Bold values indicate amino acids with significantly higher Bc^SC^ values (labeled in Figure 8B) are categorized according to their corresponding communities (see Figures 6 and 7). Community‐based functional roles, location in the structure (SSE: Secondary structure elements), related references in the literature and involvement in cancer, are also included. Shaded cells denote amino acids corresponding to unique MC/SC‐community‐based functional groups in each simulated form. (*) obtained by a search of the cBioPortal for Cancer Genomics (https://www.cbioportal.org) [36]. (a), (b): Residues with high Bc^SC^ values identified in simulated APO forms of another disease‐related kinase and of an inactive variant, respectively [47]; (c), (d), (e): Residues corresponding to conserved dynamic spine bridging side chains in the inner hydrophobic core of active APO‐, ATP‐, or ATP/substrate‐bound EPKs [43]; (f) Amino acids reported to move as a conserved “pivot block” in protein kinases [41]; (g) proposed to contribute to SRPK1 global stabilization [26]; (h) Correspond to conserved Glu‐Arg salt‐bridge forming pair of amino acids in EPKs [51]; (i) R‐spine forming side chain [42]; (j) SRPK1 residue involved in docking‐motif/docking‐groove interactions [11].

#### 3.8.1 Strong Dynamics‐Driven Allosteric Signaling Related to Substrate Specificity Is Abolished in the Presence of the PIM‐1L Peptide

As shown in Table 1, amino acids with highly significant Βc^SC^ values are organized into distinct community‐based functional groups (see also Figure 8E,F). Like in the case of the MC‐based analysis, the most represented community among the high Bc^SC^ scoring side chains in the APO simulation, is Com‐F1^SC^ (~30%; Figure 8E, *magenta*) and is divided into two functional subgroups: namely, Com‐D^CA^/Com‐F1^SC^ and Com‐F1^CA^/Com‐F1^SC^ (Table 1). The latter is the most populated and includes I193 form αΕ, P216 from the catalytic loop and the aforementioned αF residue, A541 that is adjacent to the C‐spine‐forming, M540 (Table 1). This functional classification implies an important role of these SRPK1 side chains in propagating allosteric signaling related to substrate specificity. Supporting this hypothesis, a long hydrophobic side chain at position 193 is conserved in MAP kinases and Ala at position 541 is unique to SRPKs (conversely, they correspond to small and long side chains in other EPKs). Adding to this idea, substitution of the αF short side chain of Ala at position 541 by a longer hydrophobic side chain (as in other EPKs [42]), expected from our analysis to bypass specificity/recognition, is a gain‐of‐function change resulting in melanoma, as already mentioned, whereas a splice site mutation at this position is a cancer hotspot (Table 1).

The second functional subgroup of APO Com‐F1^SC^‐mediated strong allosteric signaling includes the αE residues, V196 and G199. As deduced by their classification into the Com‐D^CA^/Com‐F1^SC^ subgroup (Table 1, *APO*), the functional role of these amino acids is most likely to serve dynamic allosteric coupling of substrate specificity with catalysis. The aforementioned melanoma‐associated splice site mutation at V196, is therefore predicted to disrupt this coupling, in perfect agreement with the crucial role of this side chain as spine‐bridging. An equally important role is therefore suggested by our analysis for Gly199. Interestingly, a glycine at this position has been also identified as an allosteric hotspot in both active APO form and inactive mutant of another disease‐associated kinase (G1010^TYK2^ in [47]). It is also noteworthy that, together with V189 (strong signaling APO main chain, Figure 8C), three out of the five Com‐F1^APO^ signaling side chains are located in αE (I193, V196, G199^CO^), suggesting a pivotal role of this helix in determining substrate‐specificity and in coupling it with C‐spine assembly and catalysis. To the best of our knowledge, such role of the αE helix has not been appreciated before.

Interestingly, none of the aforementioned Com‐F1^SC^ signaling functional subgroups is represented in the complex (Table 1; see also below), suggesting that substrate specificity is bypassed in the case of the PIM‐1L bound SRPK1.

#### 3.8.2 Allosteric Signaling Related to AL‐OUT Configuration Dominates in the Complex, Instead

The predominant allosteric hotspot of signaling side chains in the case of the simulated complex, is Com‐F^SC^ instead (35%; Figure 8F and Table 1). Notably, these include mainly polar side chains half of which are located in αF: namely, D533, S536, and T537 (Figure 8F). As deduced by the classification of these side chains in the Com‐F^CA^/Com‐F^SC^ functional subgroup (Table 1, *COMPLEX*), their substitution by hydrophobic amino acids is predicted to promote unspecific activation, and indeed it is associated with various types of cancer (Figure 8F and Table 1). Adding to this, the three aforementioned αF residues have been proposed to serve anchoring to αF of the R‐spine and of the catalytic loop in other EPKs [42]. The catalytic loop anchoring point at 537, in particular, is occupied by a universally conserved long hydrophobic side chain in other protein kinases, whereas it is a threonine uniquely in SRPKs, implying an important role of a polar side chain at this position for regulating their catalytic activity. Supporting this hypothesis, replacement of T537 by the long hydrophobic side chain of methionine, is associated with uterine and colon cancers (Figure 8F and Table 1). The Com‐F^CA^/Com‐F^SC^ functional subgroup in the complex also includes the side‐chains of E521 and R641 (Table 1). These oppositely charged residues correspond to the Glu‐Arg (conserved APE‐R) pair of amino acids, which is conserved in EPKs and the salt‐bridge between their side chains is proposed to hold an important role in regulating their catalytic activity [50]. Interestingly, the other two universally conserved residues of the APE‐motif (Ala and Pro), are not conserved in SRPKs but are replaced by a serine (S519^SRPK1^) and a leucine residue (L522^SRPK1^), instead, suggesting an important role of these side chains in SR specificity. This idea is corroborated by the fact that their replacement by the bulky hydrophobic side chain of phenylalanine, is related to various types of cancer [36].

The second Com‐F^SC^ subgroup of side chains in the complex includes V144 from the αC/β4 loop that bridges activation (AL‐OUT) with catalysis as deduced by its classification into the Com‐D^CA^/Com‐F^SC^ functional subgroup (Table 1, *complex*). Notably, V144 is the only exception to the non‐hydrophobic character of Com‐F^SC^‐mediated signaling in the complex and has been reported as part of the pivot block [41] and a spine‐bridging in other EPKs (L103^PKA^ in [43]).

#### 3.8.3 The Activation Loop (P+1 Portion) Signals Back to the Catalytic Center Through Q513 in the Case of the Apo Enzyme, a Connection That Is Lacking in the Peptide‐Bound SRPK1

The Com‐F^CA^/Com‐F^SC^ functional group of signaling side chains in the APO simulation, on the other hand, is represented by the conserved side chains of D533 and W535 from αF, and Q513 from the substrate binding P+1 portion of the AL (Table 1, Com‐F^CA^/Com‐F^SC^ group *in APO*). The role of αF as an integration motif and of the conserved aspartate D533 and W535 in particular, is well established, as already mentioned. Namely, the role of the former is to anchor the R‐spine and of the latter to anchor the AL and the αΗ helix to αF in active kinases [42]. Indeed, and further validating our results, shortening of W335 (resulting from W535 to Leu replacement) corresponds to one of the melanoma‐associated mutations described earlier, and a Leu substitution for D533 results in lung cancer (Table 1).

The APO Com‐F^CA^/Com‐F^SC^ functional classification therefore, suggests an equally important role for Q513 in activating SRPK1 and this is to anchor the AL in the active conformation and to secure its connection to the catalytic center (see also distance 5: Q513‐I214 in Figure 5B, *left panel*).

#### 3.8.4 Allosteric Signaling From the ATP‐Binding Pocket (and the Gly‐Rich Loop)

Another prevailing functional group of highly signaling side chains in the APO simulation is Com‐B^SC^. This group is composed of three N‐lobe side chains and is divided in two functional subgroups: namely, Com‐A^CA^/Com‐B^SC^ and Com‐B^CA^/Com‐B^SC^ (Table 1). The former includes the backbone oxygen of the conserved G87 from the G‐loop and the bulky hydrophobic side chain of W95 from β2‐strand, whereas the latter contains the β3 residue, V110 (Figure 8E) that immediately follows the conserved ATP‐binding lysine, K109. The function of the APO B^SC^ group of signaling side chains is therefore related to ATP‐phosphate binding and correct positioning of αC (αC‐IN) and their role as allosteric hotspots, revealed by this study, is validated by the observation that mutation of G87 or V111, results in glioblastoma or lung cancer, respectively (Table 1).

Interestingly, Com‐B^SC^ is not represented in the complex at all, and this is most probably due to quenched dynamics of the G‐loop in the PIM‐1L peptide‐bound SRPK1 (Figure S7), which in turn, is indicative of inactive kinase forms [41], as already mentioned. The APO Com‐A^CA^/Com‐B^SC^ functional subgroup appears to be replaced by two other N‐lobe subgroups in the complex, instead. Namely, the Com‐A^CA^/Com‐A^SC^ and Com‐B^CA^/Com‐C^SC^ groups (Table 1). Com‐B^CA^/Com‐C^SC^ contains solely the side chain of the αC residue, A121 (Figure 8F, Table 1), suggesting a role of this alanine residue in regulating the R‐spine assembly depending on αC‐positioning. Given the αC‐OUT conformation and the quenched dynamics of αC around position 121 in the complex (Figure S7), this functional classification suggests that an alanine residue at this position stabilizes the αC‐OUT conformation and prevents the R‐spine assembly. Supporting this idea, substitution of A121 by a glycine residue that increases the dynamics of this region, results in glioblastoma (Table 1).

The Com‐A^CA^/Com‐A^SC^ group of signaling side chains on the other hand, involves four long hydrophobic side chains of the N‐lobe β‐structure in the complex (V82, W95, V110, and V111; Table 1) that line the upper surface of the ATP‐binding pocket (Figure 8F, in red). Notably, one side chain‐member of this group is that of V110, which in the APO simulation is part of Com‐B^SC^ (Table 1) and is therefore predicted by our analysis to serve sensing correct, αC‐IN, positioning. Indeed, and as already described earlier, substitution of this valine by a longer hydrophobic side chain (Ile), predicted to enhance this signaling, is associated with lung cancer, validating our results.

#### 3.8.5 Allosteric Signaling From the Spacer Insert Region

**FIGURE 9:**
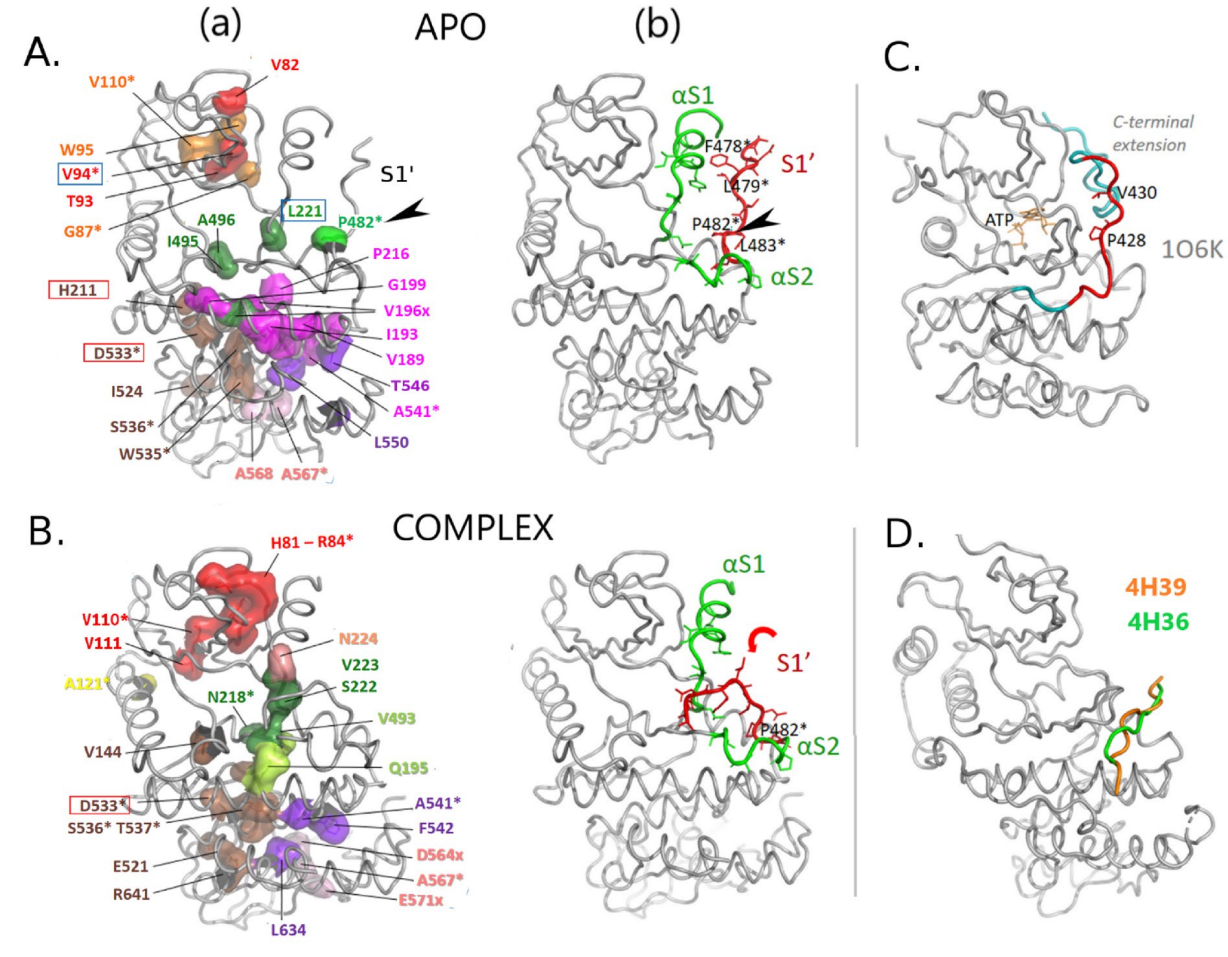
Comparison of spacer insert conformations adopted in the two SRPK1 forms simulated in this study, with other protein kinases. (A, B) MD‐snapshots of the simulated SRPK1 forms: (a) Back views of the illustrations shown in Figure 8E,F and (b) tube‐representations with highlighted the spacer region, shown side‐by‐side with the known crystal structures of other protein kinases. Namely, (C) an active ternary complex of Akt kinase (PDB ID code: 1O6K [52]) and (D) an inactive form of JNK3 kinase (assembled; unphosphorylated‐AL‐IN) in complex with peptides from its substrate ATF2 and the scaffold protein JIP1 (PDB ID codes: 4H39 and 4H36 [53], respectively). The arrow indicates the strongly signaling spacer residue, P482, identified in the APO SRPK1. This figure shows that the distinct conformations of the dynamic spacer element S1′ relative to the core of the kinase, observed in the APO and PIM‐1L peptide‐bound SRPK1, are reminiscent of intra‐molecular and docking interactions observed in other active and inactive protein kinases, respectively.

As already mentioned above, the spacer residue P482 is also found among the contributors to allosteric signal propagation in the APO SRPK1 (Figure 8B, *Left panel* and Figure 8E). Notably, P482 is located at the base of the S1′ spacer element that changes conformation in the presence of the PIM‐1L peptide (Figure 8E,F, see also Figure 9A,B). As implied by its classification in the Com‐D2^CA^/Com‐D1^SC^ functional group (Table 1, *APO*), the role of P482 is to serve allosteric communication between the lobes that in turn, is a key aspect of active kinases. This is most likely aided by increased dynamics of S1′ and of its adjacent αS2 helix in the APO form (Figure S7). These observations are in line with the idea that the spacer helices contribute to global stabilization of SRPK1 and suggest an equally important role for the dynamics of the S1’ element in stabilizing the active conformation of the enzyme. Indeed, in the APO simulation, P482 together with other hydrophobic side chains from S1′ (F478, L479, L483: labeled in Figure 9A‐b) contributes to a network of hydrophobic interactions reminiscent to those observed in other active protein kinases (Figure 9C), including SRPK1 [26]. Validating our results, a Leu substitution for P482 corresponds to one of the melanoma‐associated mutations of *SRPK1* described earlier (Table 1) and S1′ itself is a cancer hotspot (indicated with asterisks in Figure 9A‐b), as revealed by screening the cBio Cancer Genomics portal [36]. Namely, replacement of the aforementioned S1′ hydrophobic side chains by both shorter (L478V and L479V mutations) or larger ones (L479/F/I and L483I mutations), predicted to disrupt or stabilize this network respectively, results in various types of cancer. L479F in particular, is predicted as a gain of function change and interestingly it corresponds to yet another melanoma‐associated mutation of *SRPK1*, further supporting our results.

On the contrary, and as shown in Figure 9B,C, the flip of S1′ observed in the complex, mimics inhibitory protein–protein interactions observed in other kinases. For example, the mode of binding of S1′ on the back of the kinase core in the case of the complex, is reminiscent of inter‐molecular docking on yet another, opposite to the active‐center, docking groove (ED‐site) on the back of other MAP kinases (see e.g., binding of scaffold proteins in the inactive, AL‐unphosphorylated form of JNK3 kinase [53] and Figure 9D), or of intra‐molecular, auto‐inhibitory docking of regulatory modules, such as SH2‐SH3 subdomains on the back of Abl kinase that promotes inactive conformations [50]. This conformational switch of S1′ is accompanied with significantly reduced fluctuations of the C‐terminal portion of the spacer (Figure S7) and this in turn is most likely responsible for altered signaling from the spacer in the presence of the PIM‐1L peptide. Instead, allostery is now mediated only by the backbone of the region connecting αS1 with β7 (S222 to N224, Figure 9B‐a), suggesting that activating/inactivating signal transmission from the spacer depends on the presence of the docking‐groove‐bound docking peptide and vice versa.

#### 3.8.6 Allosteric Signal Transmission From the Docking Groove

Finally, three side chains from the C‐terminal end of αF (T546), the αF/αG connecting loop (L550) and αH (A623), form a functional group (Com‐H^CA^/Com‐H^SC^) related to signaling from the docking groove in the APO case (Table 1 and Figure 8E). Additional allosteric signal propagation from the docking groove is mediated by the αG side chains of A567 and I569 (Com‐G^CA^/Com‐G^SC^ in Table 1, *APO*). Actually, as illustrated in Figure 8E, signaling from the docking groove mediated by the network of hydrophobic side chains of the aforementioned αG residues and of W535, seems to contribute to the R‐spine assembly through D533 and the backbone of H211 (see also Figure S9A, *left panel*). Supporting this idea, a valine substitution for A567 that is predicted by our analysis to enhance this signal, is associated with endometrial carcinoma (Table 1). On the other hand, replacement of the bulky W535 by a the much shorter side chain of leucine, as in the melanoma‐associated W535L mutation, is therefore predicted to disrupt such signaling. Based on these results, an equally important role for the long hydrophobic αG side chain of I569, is implied by our analysis.

On the contrary, in the presence of the PIM‐1L peptide, the Com‐G^CA^/Com‐G^SC^ functional subgroup does not include I569 (Table 1) and the aforementioned mentioned allosteric signaling from the docking groove as well as the R‐spine assembly, observed in the APO case, are disrupted (Figure 8F and Figure S9B). Instead, signaling from the docking‐groove in the complex involves mainly the backbone of the αG helix at D564 to E571 (Figure 8D), the side chains of which, in turn, bind the PIM‐1L arginine residue at docking‐motif position −1 (Figure 4B).

Taken together these observations, in conjunction with the inactive αC‐OUT conformation observed in the simulated complex, suggest that binding of the docking‐motif‐like PIM‐1L peptide interferes with allosteric signaling from the docking‐groove related to the R‐spine assembly and therefore to activation. In line with this hypothesis, and validating our results, a splice‐site mutation of D564 is predicted to result in gain of function and is associated with invasive breast cancer, whereas a nonsense mutation at E571 is a cancer hot‐spot [36].

### 3.9 Quantitative Analysis of the Causality and Energetics of Allosteric Communication

An independent, complementary method to approach allostery could include quantitative (energetic) analysis of allosteric causality and modulation using the structure‐based statistical mechanical model of allostery (SBSMMA) [54, 55]. This type of analysis is based on the idea that the cause of allosteric signaling in proteins is a perturbation of their equilibrium state that results in a change of their free energy landscape. SBSMMA allows exploring the nature of causality and quantification of allosteric responses in terms of per residue free energy changes upon perturbations, such as ligand/effector binding, mutations and their combinations [54]. This in turn allows estimation of a purely allosteric effect delivered to a residue as a result of a given perturbation (P) based on the notion of the per‐residue (i) background‐free free energy change, called allosteric modulation, Δ*h*_*i*_^(P)^ (introduced in [55]). We plan to apply SBSMMA, as implemented in the AlloSigMA 2 server [56] to explore allosteric signaling modulation within SRPK1‐KD caused for example, by PIM‐1L binding and/or mutations of key amino acids identified in the present study, including cancer‐related mutations (Table 1). This is a work currently in progress and some preliminary results are shown in Table S1 and described below:

For example, as shown in Table S1, binding perturbation experiments using the first mode of operation of AlloSigMA 2 [56] and the PIM‐1L peptide‐, ATP‐ or substrate‐binding as a source of perturbation on the MD snapshots of both the APO (active) and PIM‐1L‐bound (inactive) SRPK1‐KD forms, show that binding perturbation of the docking groove (mimicking PIM‐1L peptide‐binding) induces a much larger positive allosteric modulation at the ATP‐binding site in the APO SRPK1‐KD compared to its inactive, CPLX, form (^APO^Δ*h*_ATP_^(PIM‐1L)^ = 1.22 Kcal/mol vs. ^CPLX^Δ*h*_ATP_^(PIM‐1L)^ = 0.17 Kcal/mol, Table S1). Reversely, a much weaker allosteric signal is delivered at the APO docking‐groove (PIM‐1L site) upon binding perturbation at the ATP site compared to the inactive case (^APO^Δ*h*_PIM‐1L_^(ATP)^ = 0.28 Kcal/mol vs. ^CPLX^Δ*h*_PIM‐1L_^(ATP)^ = 0.84 Kcal/mol, Table S1). Moreover, an excess of ~0.6 Kcal/mol is delivered at the APO spacer region upon binding perturbations of either the ATP‐ or PIM‐1L sites, relative to the inactive form (ΔΔ*h*_spacer_^(PIM‐1L)^ = 0.62 Kcal/mol and ΔΔ*h*_spacer_^(ATP)^ = 0.61 Kcal/mol, Table S1). It is also noteworthy that opposite ΔΔ*h*_ActiveSite_^(ATP)^ values are observed in the APO and CPLX forms, indicating that a stronger allosteric response is delivered at the active site upon ATP binding in the active relative to the inactive case (ΔΔ*h*_ActiveSite_^(ATP)^ = 0.44 Kcal/mol, Table S1). Finally, a both‐ways allosteric coupling of 0.15 Kcal/mol between the ATP‐ and substrate‐binding sites was observed in the APO SRPK1‐KD, which is disrupted in the complexed form, as indicated by zero or negative values of allosteric modulations caused by the corresponding binding perturbations (^CPLX^Δ*h*_ATP_^(SUB)^ = −0.02 and ^CPLX^Δ*h*_SUB_^(ATP)^ = −0.24; shaded cells in Table S1). Collectively, and as suggested by positive ΔΔ*h*_site_^(P)^ values in Table S1, these results suggest that the mode of action of PIM‐1L binding at the docking groove is to prevent functional sites (such as ATP‐ and substrate‐binding sites, active site) to reach their dynamic state required for allosteric activation. Interestingly, this holds also true for the spacer region. These results are in accordance with the community network analysis and betweenness‐centrality‐based results described above (Section 3.8), and combined with the increased fluctuations of the aforementioned sites in the APO form, especially of the docking groove, substrate‐binding regions and the spacer (Figure S7), further highlight the crucial role of dynamics in allosteric regulation of kinase activity. More extensive quantitative analyses using the SBSMMA protocol (e.g., as benchmarked in [57]), will be required in order to further explore the involvement of allostery in modulating SRPK1's activity.

## 4 Conclusions

In this study, using a combination of biochemical and in silico approaches, we investigated whether the SR/SH‐rich domain of the long isoform of PIM‐1 kinase could act as a substrate of SRPK1.

Our biochemical data from GST‐pull‐down and in vitro phosphorylation assays provided evidence that PIM‐1L binds to, but is not phosphorylated by SRPK1 and that the PIM‐1L SR/SH‐rich domain is indispensable for this interaction. Moreover, kinase activity assays revealed that the SR/SH‐mediated interaction inactivates SRPK1. Subsequent 3D‐modeling of a peptide from the PIM‐1L SR/SH domain in complex with SRPK1 combined with long MD simulations of both peptide‐bound and unbound (APO) SRPK1, shed light on the atomic details of this interaction and on its structural consequences on the SRPK1‐KD.

Namely, and in line with our biochemical data, the MD results showed that the PIM‐1L SR/SH region can mimic docking‐motif/docking‐groove interactions observed in other SRPK1 complexes and that this interaction is accompanied with conformational changes of SRPK1 including a swing‐OUT configuration of the αC helix, indicative of inactive kinases. Adding to this, our MD results showed that binding of the peptide into the docking groove reduces the dynamics not only of the docking groove itself but also of various distant SRPK1 regions, such as the G‐loop, the N‐terminal part of the αC helix, the αD/αE connecting loop and especially of the C‐terminal portion of the SRPK‐characteristic spacer insert. We conclude that the PIM‐1L SR/SH domain can function as a pseudo‐docking peptide for SRPK1 that acts as a switch from active (as in the APO form) to inactive (αC‐OUT) conformations and that the dynamics of the aforementioned affected SRPK1 regions is crucial for allosteric activation of this enzyme.

Subsequent comparative community map analyses of the MD trajectories unraveled the details of the dynamic architecture of the unbound SRPK1 that was unknown until now, and shed light on alterations of allosteric communications in response to PIM‐1L peptide binding. This analysis allowed us to identify networks of residues with correlated movements in each simulated form and to predict their function. More specifically, we found that the most represented community of residues in the apo enzyme is related to substrate specificity (Com‐F1), and that this community also includes conserved spine‐forming and spine‐bridging residues found in active EPKs. These results are in line with the role of the dynamics of such residues in specific allosteric kinase activation [43] and suggest that, apart from the C‐spine, the R‐spine is also sensitive to the nature of the substrate in SRPK1. This finding is in contrast to the substrate‐independent nature of the R‐spine in other EPKs and in its PIM‐1L peptide‐bound form simulated here.

Moreover, and more importantly, use of a metric based on the influence of each residue in allosteric communications (the node‐betweenness centrality measure), pointed to key SRPK1 residues with a pivotal role in mediating dynamics‐driven allosteric signaling within the kinase core both in its free form and in response to PIM‐1L peptide binding.

Validating our results, the majority of the key signaling SRPK1 amino acids identified in the present study are related to cancer‐associated mutations of the *SRPK1* gene, as revealed by screening of a portal for cancer genomics. Using community‐based functional classification, we were able to predict the role of each one of the identified signaling residues and the functional consequences of related cancer‐associated amino acid changes (Table 1), in further support of the idea that consideration of protein dynamics and elucidation of allosteric signaling offer a very powerful tool toward the identification of disease‐causing mutations (see also [47]).

Collectively, our community map analysis results suggest that the presence of the docking‐groove‐bound PIM‐1L peptide quenches the dynamics of various substrate recognition /binding/docking and specificity‐determining sites and disrupts their dynamic communication with the active site, thus interfering with specific allosteric SRPK1 activation.

More precisely, the strongest side chain‐mediated allosteric signal propagation within the APO form is related to substrate specificity‐determination and the SRPK1 αE residues, I193, V196, and G199 as well as the αF side chain of A541, along with the catalytic residue P216, hold a pivotal role in this. We were therefore able to predict, for example, the functional role of the melanoma‐associated amino acid changes at position 196 and 541 and this is interference with substrate specificity. Indeed, mutations of A541 in particular, are predicted as gain‐of‐function changes [36]. Based on the observation that such signaling is lacking in the complex, in conjunction with the SRPK unique nature of the aforementioned side chains (especially of A541 that is unique to SRPKs), we conclude that substrate specificity/recognition is bypassed in the case of the PIM‐1L bound SRPK1. In addition, these results suggest a crucial role of the SRPK1 αΕ helix in coupling substrate specificity with catalysis and thus in regulating kinase activity.

Furthermore, we found that signal propagation from the G‐loop, the C‐terminal portion of the spacer region and from the docking groove is also altered in the case of the complex. Signaling from the free docking‐groove, in particular, mediated by a network of dynamic hydrophobic interactions involving the side chain of I569, is transmitted to the R‐spine through the conserved αF aspartate residue, D535 (R‐spine regulator) and the HxD‐motif histidine (R‐spine forming). This result, in conjunction with the observation that the R‐spine is assembled in the unbound SRPK1 (despite the absence of AL‐phosphorylation), allowed us to conclude that this docking‐groove‐originated signal is essential for the stabilization of the R‐spine assembly and therefore for SRPK1 activation. On the contrary, binding of the PIM‐1L peptide (especially of docking‐motif position −1) disrupts this signaling, a result also supported by preliminary quantitative analysis of the energetics of allosteric signaling using the SBSMMA protocol. This finding offers an explanation for observations on other SRPK1 interactions reported in the literature. For example, in the case of the long RS‐domain‐containing SRPK1 substrate, ASF/SF2, the RS‐repeats themselves were found to not affect the mechanism of phosphorylation, and only the presence of the docking‐motif limited phosphorylation of this substrate [9]. Our results therefore, suggest that disruption of a dynamics‐driven allosteric signaling from the docking groove back to functional sites as observed upon binding of the docking‐motif‐like PIM‐1L peptide, may hold true for other docking‐motif/docking‐groove interactions of SRPK1.

In total, this work provides insights not only on the mechanism through which the PIM‐1L SR/SH domain inactivates SRPK1, but also adds to knowledge on the mechanisms of SRPK1 regulation, in general. Furthermore, we believe that our data may be valuable to experimental researchers for the design of new experiments toward the elucidation of PIM‐1L cellular function(s) and signaling pathways.

## Author Contributions

**Nastazia Lesgidou:** investigation, methodology, data curation, visualization, formal analysis. **Anastasia Koukiali:** methodology, investigation, data curation, formal analysis, visualization. **Eleni Nikolakaki:** conceptualization, funding acquisition, writing – original draft, writing – review and editing, supervision, methodology, data curation, formal analysis. **Thomas Giannakouros:** conceptualization, funding acquisition, writing – original draft, writing – review and editing, supervision, data curation, formal analysis. **Metaxia Vlassi:** conceptualization, methodology, investigation, data curation, formal analysis, supervision, funding acquisition, visualization, writing – original draft, writing – review and editing.

## Conflicts of Interest

The authors declare no conflicts of interest.

## Supporting information


**Data S1** Supporting Information.

## Data Availability

The data that support the findings of this study are available from the corresponding author upon reasonable request.
