# Supplementary material for: PIM‐1L Kinase Binds to and Inactivates SRPK1: A Biochemical and Molecular Dynamics Study
Source: Proteins. 2024 Oct 27;93(3):629–53. doi: 10.1002/prot.26757 (PMC11809128; doi:10.1002/prot.26757)
Supplement: Supplementary file 1 — Data S1 Supporting Information. [file PROT-93-629-s001.pdf]

Supplementary Material for

Proteins: Structure Function & Bioinformatics

doi:10.1002/prot.26757

PIM-1L kinase binds to and inactivates SRPK1: A Biochemical and Molecular Dynamics Study

Nastazia Lesgidou[1,a], Anastasia Koukiali[2,a], Eleni Nikolakaki[2], Thomas Giannakouros[2] and Metaxia Vlassi[1,*]

[1]Institute of Biosciences & Applications, National Center for Scientific Research "Demokritos", Athens, Greece

[2]Laboratory of Biochemistry, Department of Chemistry, Aristotle University, 54124 Thessaloniki, Greece

*** Corresponding author**: Metaxia Vlassi, E-mail: meta@bio.demokritos.gr

[a] authors with equal contribution: NL, AK

This file includes:

Supplementary Figures S1 to S9

and Supplementary Table S1' with their captions and references.

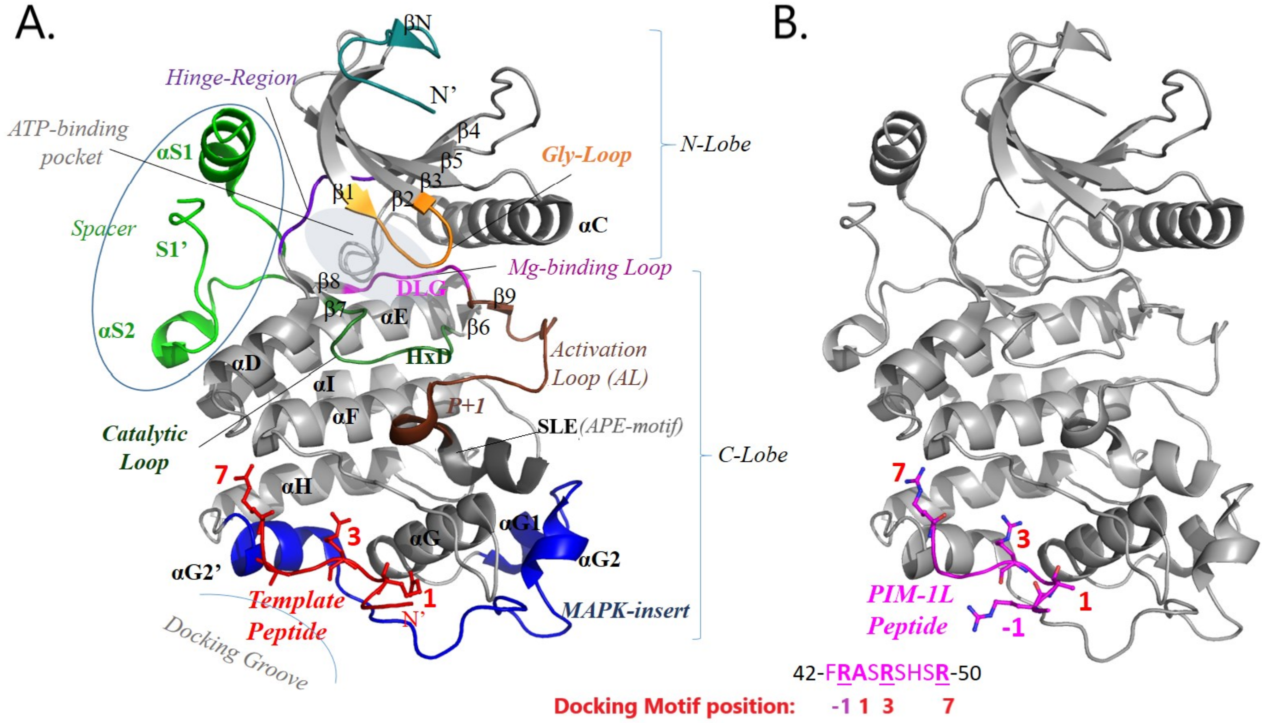

Figure S1. 3D-Modelling of the docking-motif/docking-groove interactions of a PIM-1L peptide with SRPK1, simulated in this study. (A) *Part of the known crystal structure (PDB ID: 3BEG, Ngo et al., 2008) of a complex of SRPK1 kinase domain (termed SRPK1-KD hereafter; depicted in ribbon representation) with an ASF/SF2 peptide, used as template to model the initial conformation of the SRPK1/PIM-1L peptide complex simulated in this study. SRPK1-KD adopts the conserved bilobal fold of protein kinases: namely, it is divided into two lobes, the small N-terminal lobe and a large C-terminal lobe. The adenosine-triphosphate molecule (ATP) and the substrate region to be phosphorylated, bind in the cleft between the lobes: at the ATP-binding pocket and the P+1 portion of the activation loop, respectively. SRPK1 secondary structure elements (for details see Ngo et al., 2007; Ngo et al., 2008) and important sequence motifs conserved in eukaryotic protein kinases (such as DFG-, HxD-, and APE-motifs as well as the Gly-rich-, Activation- and P+1 loops), are indicated (details on conserved motifs in: Kannan and Neuwald, 2005; Kornev et al., 2008). Note that the SRPK1-KD template structure also includes fragments of the spacer region (encircled; see also Ngo et al., 2008) and that the DFG and APE motifs are replaced by the DLG and SLE sequences, respectively, uniquely in SRPKs. The template docking peptide, with the arginine residues depicted in sticks and their corresponding docking motif positions, are shown in red.* ***(B)*** *The initial 3D-model of the PIM-1L peptide (in magenta) docked into the SRPK1-KD docking groove, produced in this study and used as starting conformation in the MD simulation of the complex. The PIM-1L peptide amino acid sequence and the corresponding docking motif positions are shown below the panel. The PIM-1L peptide was removed in the case of the SRPK1 APO form starting 3D-model.*

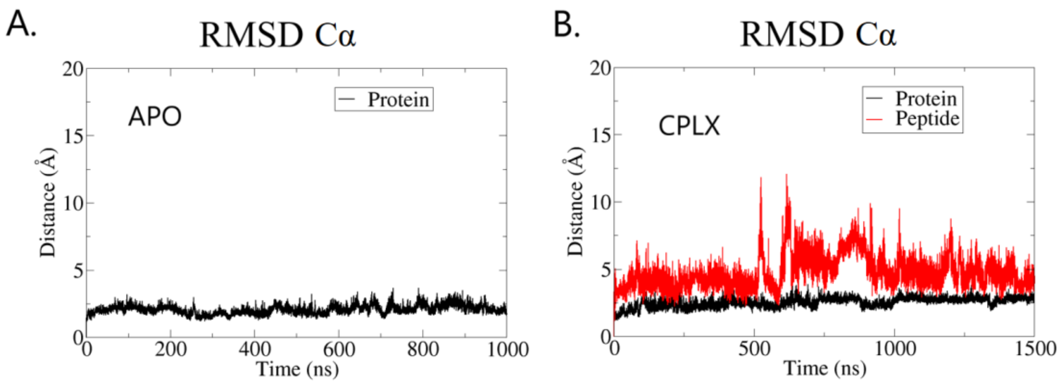

***Figure S2: Root-Mean-Square Deviation (RMSD) analysis of the MD trajectories** of **(A) the SRPK1 APO-form and (B) the SRPK1/PIM-1Lpeptide complex**, simulated in this study. Monitoring of RMSD values of SRPK1 (protein, in black) and PIM-1L peptide (peptide, in red) Cα-atoms, from their initial positions, along the corresponding MD trajectories. Both simulations had converged (RMSD values reached plateaus) in the last 500 ns of each MD trajectory and this time range was used in subsequent analyses of the corresponding MD simulations.*

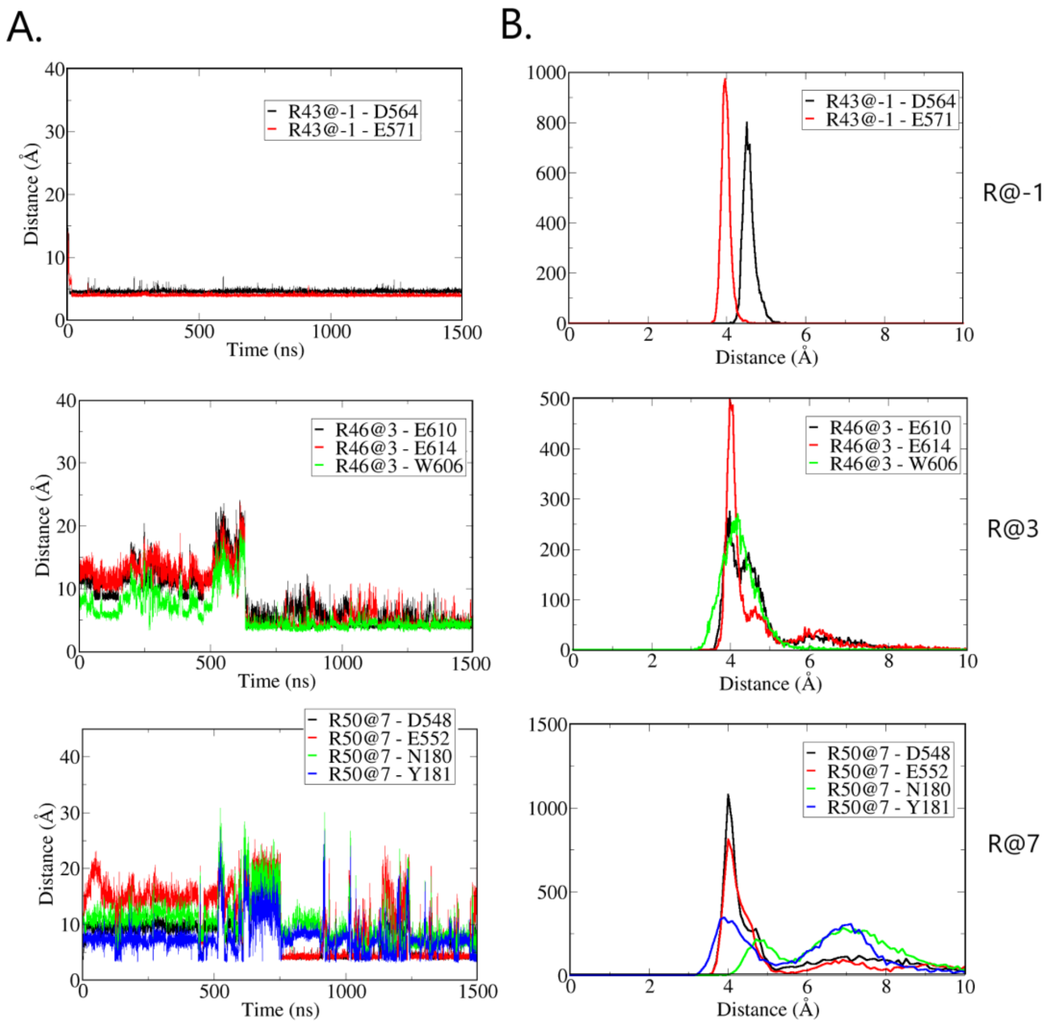

Figure S3. Monitoring of the docking-motif/docking-groove interactions of the PIM-1L peptide with SRPK1 simulated in this study. (A) *Monitoring along the entire MD trajectory and* ***(B)*** *distributions within the last 500 ns, of side chain distances of PIM-1L arginine residues at docking motif positions: -1 (upper panel), 3 (middle panel) and 7 (lower panel), with amino acids of the SRPK1 docking groove, as indicated. Once formed (distances < 5Å), the indicated groove interactions remain stable during the rest of the simulation time, strongly supporting the idea that the PIM-1L RS/RH-rich domain may target the SRPK1 docking groove and mimic docking-motif/docking-groove interactions used for other SRPK1 protein partners (substrates/inhibitors).*

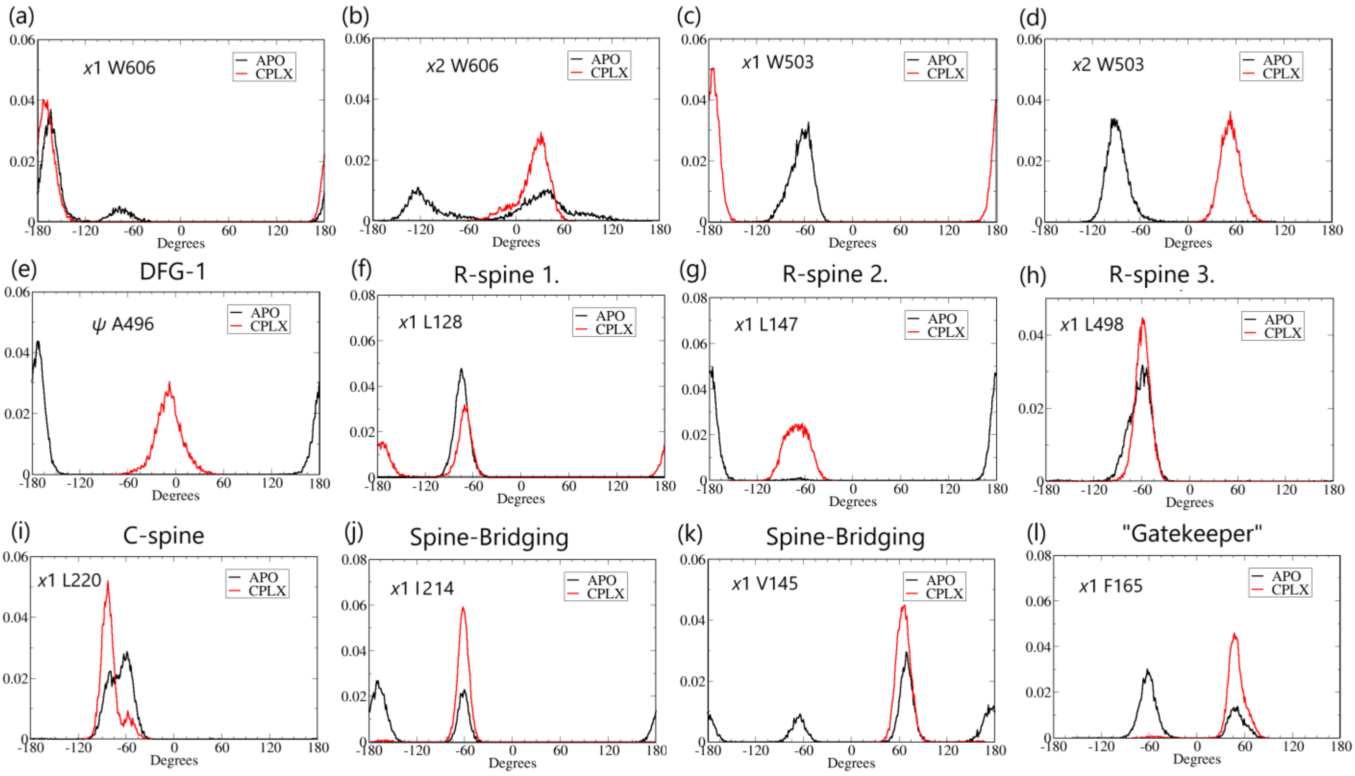

Figure S4. Dynamics of important hydrophobic amino acids of the SRPK1 APO- and PIM-1L peptide-bound forms. *Comparative plots for various backbone (ψ) and side chain (χ1,χ2) dihedral angles of critical residues of the inner-hydrophobic core of the APO (black curves) and peptide-bound SRPK1 (CPLX; red curves), as obtained by angle distribution calculations over the last 500 ns of the corresponding MD trajectories. Multi-peak χ1 and/or χ2 distributions indicate highly dynamic side chains. Overall, altered dynamics are observed in the peptide-bound form for various side chains, including those corresponding to the conserved hydrophobic R-, C-spine and R-shell forming amino acids (Kornev et al., 2008). In addition, increased side chain dynamics are detected for spine-bridging side chains (including the "gatekeeper") in the simulated APO form as compared to the complexed one, in line with their reported role in serving dynamics-driven allosteric activation of protein kinases (Kim et al., 2017) and suggestive of an inactive SRPK1 form upon binding of the PIM-1L peptide.*

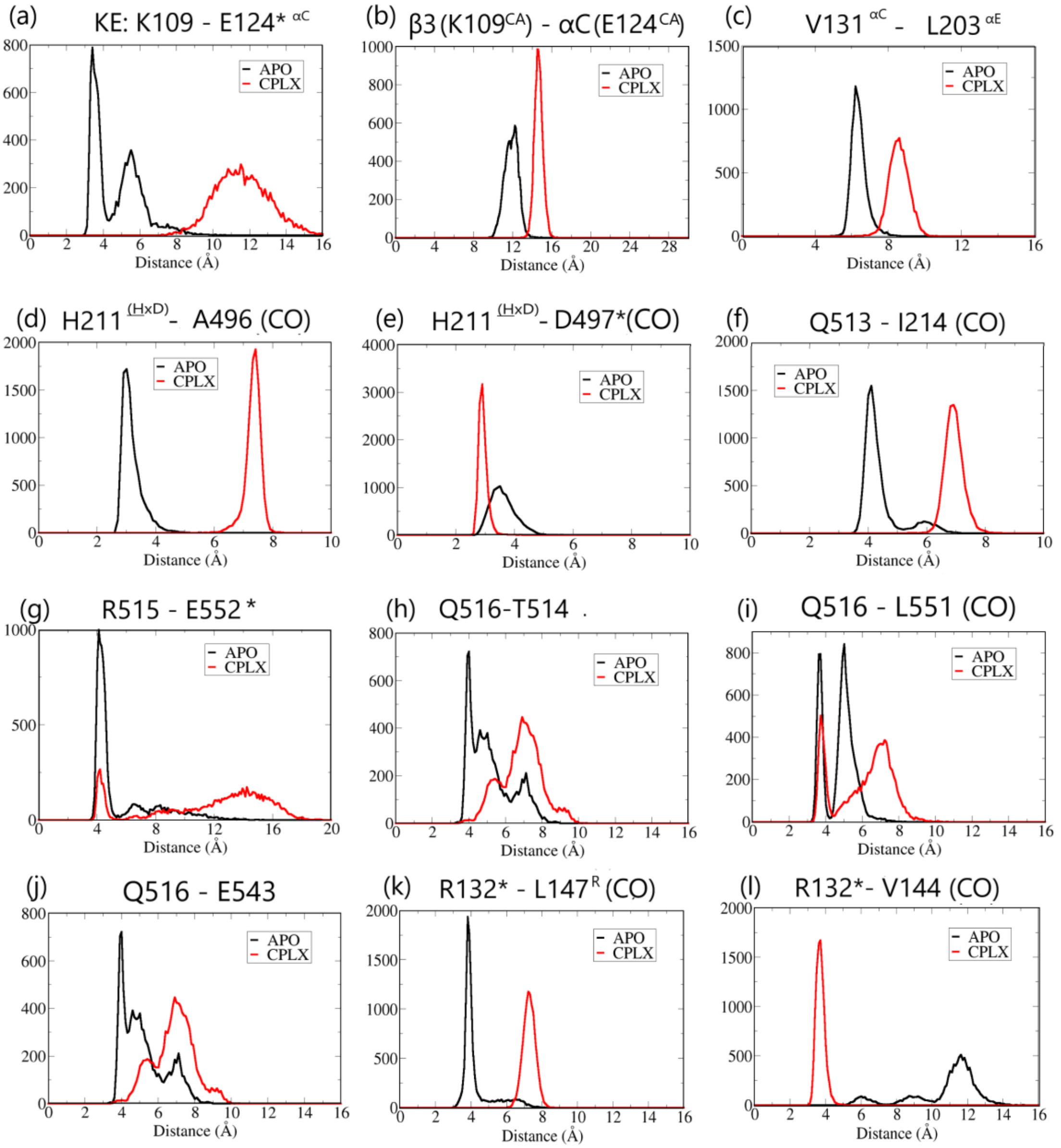

Figure S5. Comparative distribution plots of intra-KD interactions of SRPK1 in its APO- and PIM-1L peptide-bound forms. *Distributions of the indicated distances of SRPK1 amino acids (corresponding to intramolecular interactions shown in Fig. 5), over the last 500 ns of the corresponding MD trajectories, are plotted as in Fig. S4. Side chain atoms are taken into account, unless noted otherwise (CO labeling indicates backbone oxygen-atoms). As reflected by altered distance distribution profiles, binding of the PIM-1L peptide results in the disruption (distances >> 6 Å; CPLX case) or altering (compare panels k and l), of strong interactions detected in the APO SRPK1 simulation (at hydrogen-bonding distances: ≤ 4Å; APO case).*

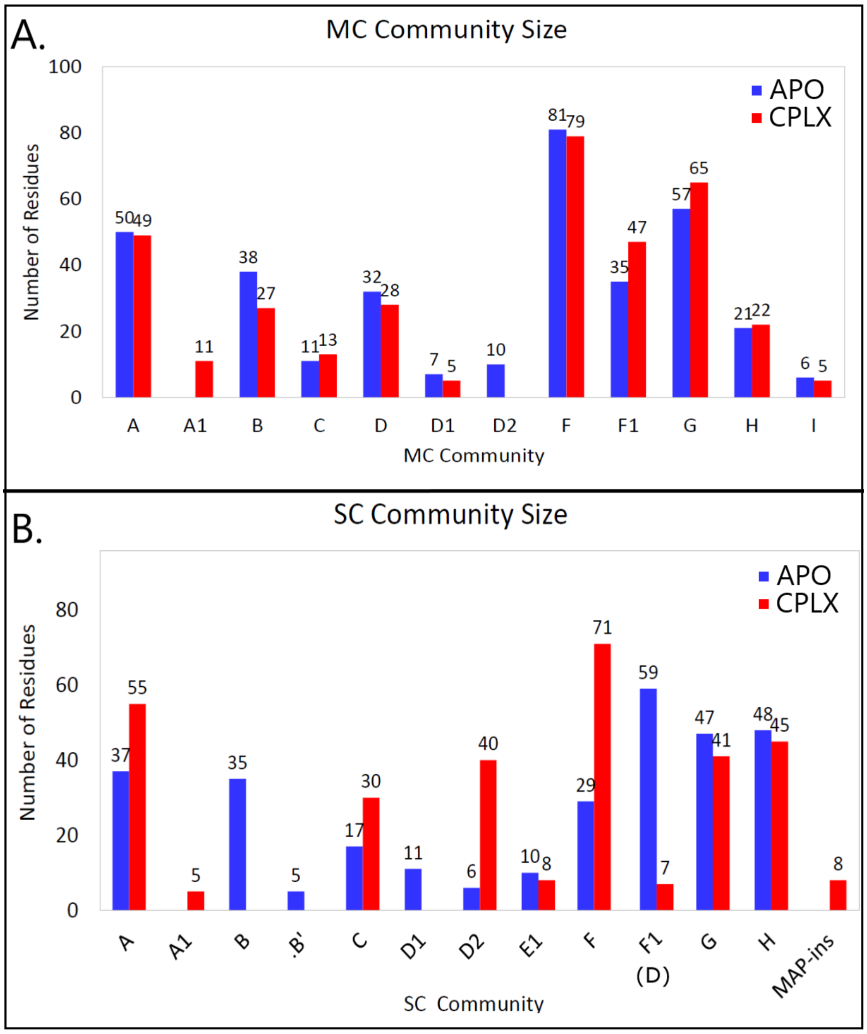

Figure S6. Comparative community-size plots. *Number of residues of* ***(A)*** *main chain, MC- and* ***(B)*** *side chain, SC-membered communities of SRPK1 in the APO form (blue bars) and in complex with the PIM-1L peptide (red bars), shown in Fig. 6 and Fig. 7, respectively. Community D is a solely main-chain community, although* $F1^{SC}$ *can be also considered as the* D^{SC} *community instead (see text). Three extra, solely SC-membered communities are detected: E1 (in both simulated SRPK1 forms), B' (in the APO form) and MAP-ins (in the PIM-1L peptide-bound form). Significant community-size differences between the simulated SRPK1 forms are observed in the case of the SC communities that are more pronounced for communities* $F1^{SC}$ *and* B^{SC} *in APO (+52 and +35 residue members), and* F^{SC} *and* $D2^{SC}$ *(+42 and +34 residue members) in the complex simulations, respectively.*

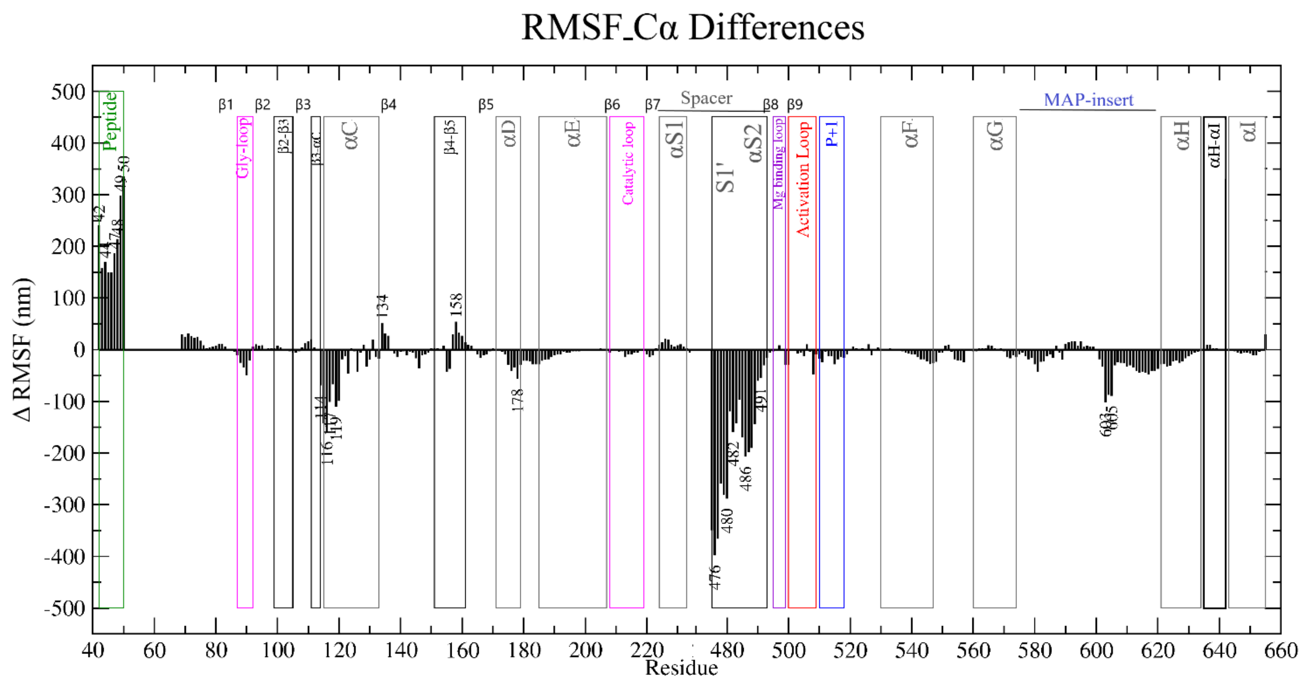

Figure S7. Cα-atom root-mean-square fluctuation (RMSF) differences between the two SRPK1 forms simulated in this study, plotted along the amino acid sequence. *Cα RMSF values for each simulated system were averaged within the last 500 ns of the corresponding MD trajectory and differences of RMSF values were calculated from: ΔRMSF = RMSF(Complex) – RMSF(APO). Negative ΔRMSF values indicate higher backbone fluctuations in the simulated APO-SRPK1 vs the SRPK1/PIM-1L peptide complex, and vice-versa. SRPK1 secondary structure elements and various regions corresponding to characteristic EPK features, are boxed and labeled (refer also to Fig. S1A, for comparison). This figure reveals that binding of the PIM-1L peptide is accompanied with significantly reduced fluctuations of various SRPK1 regions such as the G-loop, the N-terminal half of helix αC, the αD/αE connecting loop and especially the docking groove (e.g. aF/aG loop and MAP kinase insert) and the C-terminal portion of the spacer insert (S1' and aS2).*

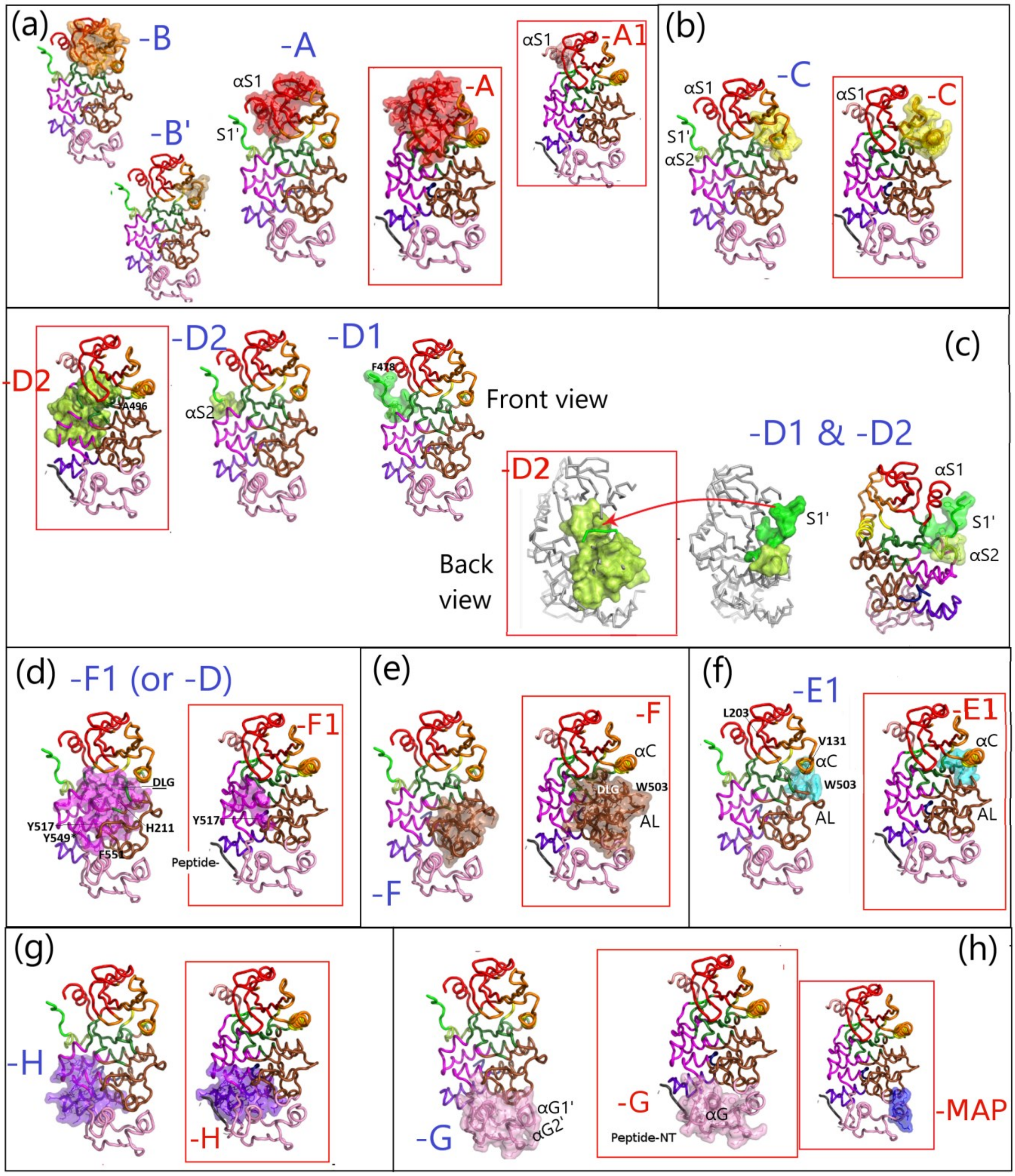

Figure S8. Comparative surface representations of side chain communities *of the APO- (blue labeled) and PIM-1Lpeptide-bound (red-boxed and red-labeled) SRPK1 forms simulated in this study.* ***(a)-(h)*** *Identified SC-communities (shown in Fig. 7A-B,a) are mapped independently and are shown in surface representation of their corresponding residue-members and side-by-side for the two simulated SRPK1 forms, for comparison. Coloring of community surfaces is as in Fig. 7, whereas the SRPK1 backbone is rendered in tube-representation and colored according to the corresponding MC-communities (as in Fig. 6). The view is as in Fig. S1, with the exception of panel c, where a back view is also shown to better illustrate the flip of the S1' spacer element observed in the complex compared to the APO case. Refer to Fig. S6B for SC-community sizes.*

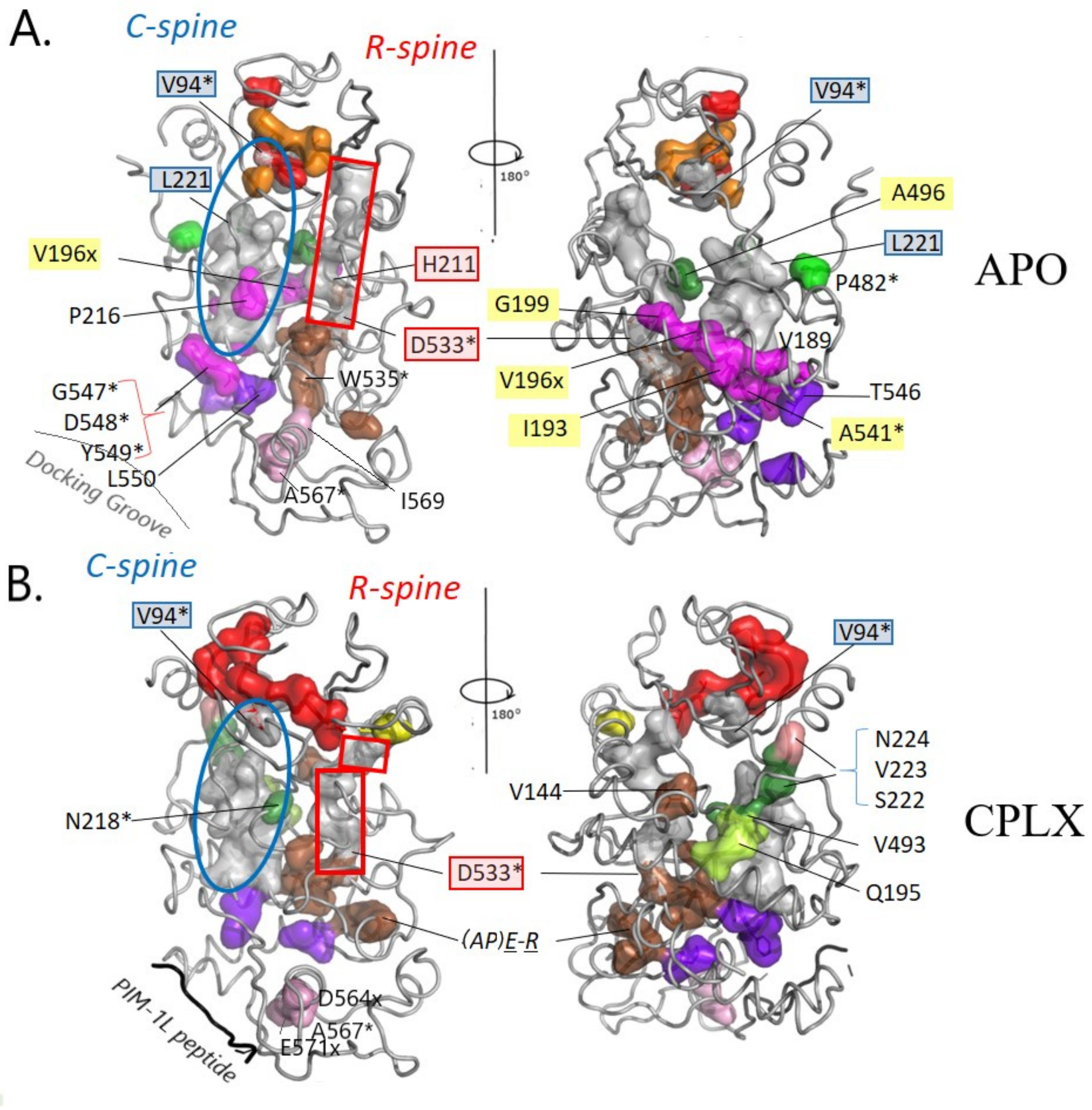

Figure S9. Strongly signaling amino acids identified in this study are shown together with the universally conserved R- and C-spine forming amino acids in the simulated SRPK1 forms. (A, B) *Front- and back-views of maps of strongly signaling residues shown in Fig. 8E and 8F (colored surfaces) are depicted along with R-spine (red-boxed) and C-spine (blue-boxed) forming ones (grey surfaces). Yellow-highlighted are selected spine-bridging residues in the APO form, discussed in the text. A network of hydrophobic side chains mediating allosteric signal transmission from the docking-groove and involving the αG residues, A567 and I569 and the aF conserved tryptophan residue, W535, seem to contribute to stabilizing the R-spine assembly in the APO case (through D533 and H211). This network of signaling side chains is disrupted in the presence of the docking-groove-bound peptide, in the complex.*

Table S1'. Allosteric responses delivered at the indicated SRPK1 sites upon binding perturbations (P) on both the SRPK1-KD forms of the present study, expressed in terms of **per-site allosteric modulations, $\Delta h_{site}^{(P)}$** (in Kcal/mol)*, calculated by applying the SBSMMA protocol as implemented in the AlloSigMA 2 server *(Tan et al., 2020*).

**Source of Perturbation (P)->**	Binding@ Docking Groove (PIM-1L site)			Binding@ATP-binding site (includes the G-loop)			Binding @Substrate-binding site, SUB[(a)]		
	$\Delta h_{site}^{(P)}$ (Kcal/mol)*								
**Modulated Site**	APO	CPLX	ΔΔh (APO-CPLX)	APO	CPLX	ΔΔh (APO-CPLX)	APO	CPLX	ΔΔh (APO-CPLX)
ATP site	**1.22**	0.17	**1.05**	-1.51	-2.63	**1.12**	0.15	-0.02	**0.17**
PIM-1L site	-2.52	-1.60	-0.92	0.28	**0.84**	-0.56	0.10	0.19	-0.09
SUB site[(a)]	0.05	-0.26	**0.31**	0.15	-0.24	**0.39**	-0.60	-0.50	-0.10
Spacer region	**1.08**	0.45	**0.62**	**0.42**	-0.18	**0.61**	0.02	0	0.02
Active site[(b)]	0.05	-0.07	0.12	**0.22**	-0.22	**0.44**	-0.60	-0.32	-0.29

** $\Delta h_{site}^{(P)}$ values were calculated using equations 8 and 10 of Guamera and Berezovsky (2019), and the per-residue free energy values (ΔG) obtained using the first mode of operation of AlloSigMA 2 (at http://allosigma.bii.a-star.edu.sg) on the MD snapshots of the APO (active) and complexed (CPLX, inactive) SRPK1-KD forms of the present study, for each indicated source of perturbation. High positive ΔΔh values indicate stronger allosteric modulations (increased dynamics of the corresponding sites) in the active relative to the inactive form. The opposite holds for negative ΔΔh values.*

[(a)] defined by similarity, based on the crystal structure of a ternary complex of an activated Akt kinase, PDB ID:1O6K (Yang et al, 2002).

[(b)] includes P+1 amino acids Q513-T514 and the catalytic base, D213

References for the Supplementary Material

Guarnera E, Berezovsky IN. Toward Comprehensive Allosteric Control over Protein Activity. Structure. 2019 May 7;27(5):866-878.e1. doi: 10.1016/j.str.2019.01.014. Epub 2019 Feb 28. PMID: 30827842.

Kannan NK, Neuwald AF. Did protein kinase regulatory mechanisms evolve through elaboration of a simple structural component? J Mol Biol. 2005;351(5):956-72. doi: 10.1016/j.jmb.2005.06.057. PMID: 16051269.

Kim J, Ahuja LG, Chao FA, Xia Y, McClendon CL, Kornev AP, Taylor SS, Veglia G. A dynamic hydrophobic core orchestrates allostery in protein kinases. Sci Adv. 2017;3(4):e1600663. doi: 10.1126/sciadv.1600663.

Kornev AP, Taylor SS, Ten Eyck LF. A helix scaffold for the assembly of active protein kinases. Proc Natl Acad Sci U S A. 2008;105(38):14377-82. doi: 10.1073/pnas.0807988105.

Ngo JC, Gullingsrud J, Giang K, Yeh MJ, Fu XD, Adams JA, McCammon JA, Ghosh G. SR protein kinase 1 is resilient to inactivation. Structure. 2007;15(1):123-33. doi: 10.1016/j.str.2006.11.011. PMID: 17223538.

Ngo, J.C., Giang, K., Chakrabarti, S., Ma, C.T., Huynh, N., Hagopian, J.C., Dorrestein, P.C., Fu, X.D., Adams, J.A. and Ghosh, G. A sliding docking interaction is essential for sequential and processive phosphorylation of an SR protein by SRPK1. Mol. Cell 2008; 29: 563-576. doi: 10.1016/j.molcel.2007.12.017.

Tan ZW, Guarnera E, Tee WV, Berezovsky IN. AlloSigMA 2: paving the way to designing allosteric effectors and to exploring allosteric effects of mutations. Nucleic Acids Res. 2020 Jul 2;48(W1):W116-W124. doi: 10.1093/nar/gkaa338. PMID: 32392302; PMCID: PMC7319554.

Yang J, Cron P, Good VM, Thompson V, Hemmings BA, Barford D. Crystal structure of an activated Akt/protein kinase B ternary complex with GSK3-peptide and AMP-PNP. Nat Struct Biol. 2002;9(12):940-4. doi: 10.1038/nsb870. PMID: 12434148.
